# Cost‐Effectiveness of Preemptive Plerixafor Versus Rescue Plerixafor for Mobilization and Collection of Hematopoietic Stem Cells in Patients With Multiple Myeloma and Lymphoma

**DOI:** 10.1002/jca.70026

**Published:** 2025-05-03

**Authors:** Roselene Mesquita Augusto Passos, Miriam Allein Zago Marcolino, Júlia Augusto Passos, Vinicius Fernando Calsavara, Leila de Lourdes Martins Perobelli, Alessandro Gonçalves Campolina, Cesar de Almeida‐Neto

**Affiliations:** ^1^ Departamento de Transplante de Medula Óssea e Hematologia Hospital de Transplantes Euryclides de Jesus Zerbini São Paulo Brazil; ^2^ Programa de Pós‐Graduação em Ciências Médicas da Faculdade de Medicina Universidade de São Paulo São Paulo Brazil; ^3^ Programa de Pós‐Graduação em Epidemiologia Universidade Federal Do Rio Grande Do Sul Porto Alegre Rio Grande do Sul Brazil; ^4^ Instituto para Avaliação de Tecnologia Em Saúde ‐INCT/IATS (CNPQ 465518/2014‐1) Universidade Federal do Rio Grande do Sul Rio Grande do Sul Brazil; ^5^ Faculdade de Medicina de Jundiaí São Paulo Brazil; ^6^ Department of Computational Biomedicine Cedars‐Sinai Medical Center Los Angeles California USA; ^7^ Centro de Investigação Translacional em Oncologia, Instituto do Câncer do Estado de São Paulo São Paulo Brazil; ^8^ Departamento de Aféreses da Fundação Pró‐Sangue Hemocentro de São Paulo São Paulo Brazil

**Keywords:** AMD3100, autologous transplantation, cost effectiveness ratio, granulocyte colony‐ stimulating factor, leukapheresis

## Abstract

**Background:**

Plerixafor combined with granulocyte colony‐stimulating factor (G‐CSF) has shown superior efficacy in mobilizing hematopoietic stem cells (HSCs). However, its widespread use is constrained by high costs, and there is ongoing debate regarding the effectiveness of mobilization strategies. This study evaluated the cost‐effectiveness of preemptive versus rescue plerixafor in patients from the Brazilian Public Health Care System with multiple myeloma or lymphoma eligible for autologous stem cell transplantation (ASCT).

**Methods:**

This observational study assessed the costs and clinical outcomes of preemptive and rescue plerixafor strategies. The incremental cost‐effectiveness ratio (ICER) was calculated for the percentage of patients with successful optimal or minimal HSC collections, who underwent ASCT and the number of leukapheresis sessions.

**Results:**

The study included 285 patients, 82 in the preemptive and 203 in the rescue group. Preemptive plerixafor resulted in a lower mobilization failure rate, a decreased need for remobilization, a higher proportion of patients progressing to ASCT, and a shorter interval between the beginning of mobilization and ASCT. The incremental cost of preemptive versus rescue plerixafor was US$ 1532.44. The incremental effectiveness observed was 10.1% for minimally successful harvest (ICER US$ 151.28), 4.7% for optimal harvest (ICER US$ 326.05), and 13.1% for patients progressing to ASCT (ICER US$ 116.18). Regarding the number of leukapheresis sessions, preemptive plerixafor was dominated.

**Summary:**

Preemptive plerixafor is a cost‐effective strategy compared to rescue plerixafor, offering higher efficacy and lower ICER values, making it a clinically beneficial option despite its higher cost.

## 1 Introduction

High‐dose chemotherapy followed by autologous stem cell transplantation (ASCT) has become the standard treatment for multiple myeloma (MM), Hodgkin's lymphoma (HL), and non‐Hodgkin's lymphoma (NHL) [1, 2, 3, 4]. The successful execution of ASCT requires the mobilization of Hematopoietic Stem Cells (HSCs), with peripheral blood (PB) being the most utilized source. A minimum collection threshold of 2 × 10^6^ CD34+ cells/kg is essential to ensure adequate neutrophil and platelet engraftment post‐transplant. Collections exceeding 4–5 × 10^6^ CD34+ cells/kg have been correlated with faster engraftment, reduced transfusion requirements, and fewer complications related to prolonged pancytopenia [5, 6].

The predominant strategy for mobilizing CD34+ cells from the bone marrow to the PB involves granulocyte colony‐stimulating factor (G‐CSF), either alone or combined with chemotherapy, with mobilization failure rates approximating 30% [7, 8, 9]. In 2008, the American Federal Drugs Agency (FDA) approved plerixafor, a selective antagonist of the CXCR4 chemokine receptor, for autologous HSC mobilization in patients with MM and NHL [10]. When combined with G‐CSF, plerixafor reduces the mobilization failure rate to less than 10%. Despite these benefits, the high cost of plerixafor limits its routine use, and a standardized practice has not yet been established [11, 12, 13].

Clinical practice remains heterogeneous, with an ongoing debate about the efficacy of remobilization strategies versus preemptive intervention for candidates exhibiting low PB CD34+ cell levels. In the Brazilian Unified Health System (*Sistema Único de Saúde*—SUS), plerixafor is not routinely included due to its high cost. However, some Brazilian SUS hospitals acquire the medication independently. Our institution typically adopts a rescue plerixafor strategy due to limited stocks, which can delay remobilization and postpone ASCT [14, 15]. Among other options, preemptive plerixafor is a strategy that uses clinical criteria to reserve the drug only for patients showing early signs of failure in mobilization. This approach has shown to be an effective intervention without excessive additional costs, gaining acceptance in clinical practice [16].

The present study evaluated the cost‐effectiveness of two plerixafor mobilization strategies—preemptive plerixafor versus rescue plerixafor. The assessment focuses on clinical outcomes such as the percentage of patients achieving minimum and optimal stem cell collections, progression to ASCT, and leukapheresis session numbers in MM and lymphoma patients eligible for ASCT.

## 2 Materials and Methods

This observational, longitudinal, and ambispective study represents a full economic evaluation in the form of a cost‐effectiveness analysis, conducted at two hospitals in the Southeast region of Brazil. The Ethics Committees of the Hospital de Transplantes Euryclides de Jesus Zerbini/Hospital Brigadeiro (HTEJZ/HB) and the Hospital das Clínicas da Universidade de São Paulo approved the study. Informed consent was obtained from prospective participants, while it was waived for the retrospective group.

Retrospectively from December 2016 to February 2019, and prospectively from March 2019 to August 2021, data were extracted from electronic medical records of patients with MM, HL, and NHL candidates for ASCT, aged ≥ 18 years, who underwent HSC mobilization with G‐CSF alone or with plerixafor. Exclusion criteria included patients with positive HIV serology, chemomobilization, leukapheresis performed with other equipment that is not Fresenius Kabi AG, Spectra Optia from Terumo or Cobe Spectra from Terumo BCT, and those whose mobilization and/or collection was interrupted due to complications (fever, bacteremia, disease progression, or equipment issues).

### 2.1 Mobilization Strategies

Patients underwent one of two mobilization strategies, preemptive or rescue plerixafor, based on the availability of plerixafor in the pharmacy stock. If at least two vials were available, patients would receive preemptive plerixafor; otherwise, they received rescue plerixafor. Patients' characteristics did not influence this allocation. The same criteria were applied to prospectively and retrospectively analyzed patients, utilizing information from the hospital's purchasing, stock department, and outpatient clinic records. In both strategies, a maximum of two vials of plerixafor were allocated per patient. The dosage was standardized at 0.24 mg/kg of actual body weight, with a reduced dose of 0.12 mg/kg administered for patients with a creatinine clearance of < 50 mL/min. The patients were hospitalized for plerixafor administration, to be performed at 11 PM, and remained hospitalized until the end of the collection process. The dosage of G‐CSF in both strategies was calculated as 20 mcg/kg/day (based on actual body weight), but it was sometimes reduced to avoid wasting of G‐CSF vials (ranging from 15 to 20 mcg/kg/day). The application was 7.5–10 mcg/kg BID in the first three days. After, the full dosage was administered, once a day, 4 h before the CD34+ cell count in PB (in the 4th day) and 4 h before leukapheresis start (in the 5th and/or 6th days).

#### 2.1.1 Preemptive Plerixafor Strategy

HSC mobilization involved administering subcutaneous G‐CSF (15 to 20 mcg/kg/day). On the fourth day of mobilization, the peripheral CD34+ cell count was evaluated. If the count was ≥ 10/μL, leukapheresis commenced the following morning and was repeated until a minimum collection of 2 × 10^6^ CD34+/kg was achieved, in up to two sessions. If the first leukapheresis yielded < 1.5 × 10^6^ CD34+/kg or if the PB CD34+ cell count was < 10/μL on day 4 (D4), G‐CSF administration continued daily, and the patient received plerixafor subcutaneously at 11 PM on the same day. Leukapheresis started approximately eleven hours after plerixafor administration and was repeated until a minimum collection of 2 × 10^6^ CD34+/kg was attained, in up to two sessions. If the final leukapheresis count over two days was < 2 × 10^6^ CD34+/kg, it was deemed a mobilization failure. Figure S1 summarizes the preemptive plerixafor strategy.

#### 2.1.2 Rescue Plerixafor Strategy

In the same manner, as in the previous strategies, HSC mobilization involved subcutaneous administration of G‐CSF (15 to 20 mcg/kg/day) for four days. On D4, if the PB CD34+ cell count was ≥ 10/μL, leukapheresis began the following day and continued daily until a minimum collection of 2 × 10^6^ CD34+ cells/kg was attained, in up to two sessions. Physicians sometimes increased the G‐CSF dosage to 20 mcg/kg/day on D4 (only for patients receiving smaller doses), and a new PB CD34+ count was performed on D5. Mobilization was considered unsuccessful if the CD34+ count on D4 or D5 was < 10/μL or if a minimum collection was not performed in up to two leukapheresis sessions. Patients failing initial mobilization with G‐CSF alone underwent remobilization after acquiring plerixafor. The second mobilization followed the intervention group's protocol with G‐CSF plus preemptive plerixafor. Figure S2 summarizes the rescue plerixafor strategy.

### 2.2 Outcomes

#### 2.2.1 Clinical Outcomes

Primary clinical endpoints included the total CD34+ HSC (×10^6^/kg) during mobilization and remobilization if needed, the percentages of patients with minimum and optimal collections, the number of leukapheresis sessions, percentages of patients requiring HSC remobilization, the time between first mobilization and remobilization (days), the percentage of patients undergoing ASCT, and the interval between first mobilization and transplantation (days). Mobilization failure was defined as a collection of < 2 × 10^6^ CD34+ cells/kg over 1–2 consecutive days of leukapheresis or a CD34+ cell count in PB of < 10/μL on D4 or D5 of mobilization. Minimum collection was > 2 × 10^6^ CD34+ cells/kg and optimal collection was ≥ 4 × 10^6^ CD34+ cells/kg up to two leukapheresis sessions.

Secondary clinical outcomes included the number of HSCs infused during transplantation, time to neutrophil and platelet engraftment, hospital length of stay (LoS), and blood product transfusions during transplantation. Neutrophil engraftment was defined as initiating three consecutive days with a neutrophil count exceeding 500 × 10^6^/L. Platelet engraftment was defined as the first of three consecutive days with a platelet count of 20 000/μL or higher, without any need for platelet transfusion for at least seven consecutive days.

#### 2.2.2 Economic Outcomes

The direct medical costs were estimated from the first consultation for HSC mobilization until the completion of HSC collection through leukapheresis: laboratory tests, transfusions of blood products, stay in the day hospital and hospital admission, mobilization, collection, and cryopreservation of HSCs, medical and nursing consultations during HSC mobilization, catheter placement, and medications filgrastim (G‐CSF) and plerixafor. Using the micro‐costing technique, the resources used by each patient were accounted for and multiplied by the unit price, thus calculating the total average cost per patient.

All costs, except for medications, were obtained from the Management System of the Table of Procedures, Drugs, Orthoses, Prostheses, and Special Materials of the SUS (Sistema de Gerenciamento da Tabela de Procedimentos, Medicamentos e OPM do SUS—SIGTAP) in February 2024. The cost of G‐CSF (filgrastim) was retrieved from the Health Prices Database (Banco de Preços em Saúde—BPS). The results reflect the weighted average of purchases over the last 18 months (August 9, 2022, to February 9, 2024). Since there were no purchase records in the BPS for the previous 18 months, the February 2024 Drug Market Regulation Chamber (Câmera de Regulação do Mercado de medicamentos—CMED) table price, reflecting the factory price exclusive of the State Value Added Tax (Imposto sobre Circulação de Mercadorias e Serviços—ICMS), was used for plerixafor.

All costs are presented in US dollars (USD), converted from Brazilian Reais (BRL) for the year 2024, using a cost conversion calculator based on purchasing power parity, available online (https://eppi.ioe.ac.uk/costconversion/). Table S1 presents the list of all resources and respective standard costs.

### 2.3 Incremental Cost‐Effectiveness Ratio

The ICER for preemptive plerixafor versus rescue plerixafor was calculated using the formula:

$$\text{ICER}=\frac{\text{Preemptive plerixafor cost}-\text{Rescue plerixafor cost}\;}{\text{Preemptive plerixafor outcome}-\text{Rescue plerixafor outcome}}$$

The average results of the groups were used for continuous costs and clinical outcomes. For categorical outcomes, the ICER was calculated per additional percentage of patients achieving the outcome.

### 2.4 Subgroup Analyses

Given that HSC mobilization may vary depending on the underlying disease, we conducted a separate analysis for MM and lymphomas. We also conducted a subgroup analysis including only patients with poor mobilization (defined as those with a CD34+ PB D4 < 10 cells/μL) overall and stratified by MM and lymphoma.

### 2.5 Apheresis

The CD34+ HSC quantification via flow cytometry was conducted at the Hospital das Clínicas (Faculty of Medicine of Sao Paulo) cell cryopreservation laboratory from December 2016 to February 2019 using the FACSCalibur—BD Flow Cytometer, and from March 2019 to August 2021 at the Sollutio Diagnostics laboratory, using the FACSCanto II—BD Flow Cytometer.

HSC collection was performed through leukapheresis using one of the following cell separation devices: at HCFMUSP, from 2016 to 2018, collections were conducted using either the COM.TEC OP‐PT (Brazil), manufactured and distributed by Fresenius Kabi AG, or the Spectra Optia from Terumo BCT. From 2019 to 2021, collections at HTEJZ/HB were performed using the Cobe Spectra from Terumo BCT. Central venous catheter implantation was performed on all patients in both groups who did not have adequate peripheral venous access. Each leukapheresis session processed between two to five blood volumes of the patient, calculated based on weight, height, and gender. The first apheresis processed four to five blood volumes, while the second apheresis processed only the blood volume needed to complete the target collection (minimum of 2 × 10^6^ CD34+/kg), ranging two to five volumes. The cryopreservation laboratory used dimethyl sulfoxide at a 10% concentration, with the bags stored in a freezer at −80°C for up to 72 h after leukapheresis. The collection efficiency coefficient (CEC) was calculated using the following formula [17]:

$$\mathrm{CEC}=\frac{\mathrm{CD}34+\text{yield}\;\left(\times \frac{10^{6}}{\mathrm{kg}}\right)\times \text{weight}\;\left(\mathrm{kg}\right)\;}{\text{collection volume}\;\left(L\right)\times \text{peripheral blood}\;\mathrm{CD}34+\text{count}\;\left(\mathrm{\mu L}\right)\times 10}$$

### 2.6 Uncertainty Analysis

Deterministic and probabilistic sensitivity analyses were performed to assess the robustness of the results and to account for variability in key parameters.

In the univariate deterministic sensitivity analysis, the parameters varied included the total average cost per patient and clinical outcomes, both adjusted using the 95% confidence interval (CI) limits. The minimum and maximum values for filgrastim were considered. Tornado charts were used to present the results.

For probabilistic analysis, we used the bootstrap methodology to assess the uncertainty of the calculated ICERs: 10000 resamplings with replacement were performed from the original data, and the ICER was calculated for each resampling, along with the confidence interval calculation.

### 2.7 Statistical Analysis

A descriptive analysis of all variables was conducted. Absolute and relative frequencies were provided for qualitative variables, while measures of central tendency and dispersion such as mean, median, minimum, maximum, and standard deviation were used for quantitative variables. The Chi‐square test or Fisher's exact test was applied to assess associations of independent qualitative variables with the group variable. The Mann–Whitney test or Student's *t*‐test was used for quantitative variables. The Shapiro–Wilk test was employed to test the hypothesis of data normality. In the case of data non‐normality, the non‐parametric Mann–Whitney U test was applied. All analyses used a bilateral hypothesis test with a significance level of 5% (*p* < 0.05). Data analysis was performed using R software version 4.0 (R Core Team. 2020).

## 3 Results

A total of 293 hematopoietic stem cell mobilizations were performed between December 2016 and August 2021. After applying exclusion and allocation criteria, 82 patients were analyzed in the preemptive strategy group, and 203 patients were analyzed in the rescue strategy group. Patient selection is depicted in Figure S3.

The retrospective and prospective cohort distribution was similar between the two mobilization strategy groups. In the preemptive strategy group, 52 (63.4%) patients were prospectively evaluated compared to 118 (58.1%) in the rescue plerixafor strategy group (*p* = 0.49).

### 3.1 Patient Characteristics

Patient characteristics are described in Table 1. The diagnosis, disease stage, and prior treatments for MM, HL, and NHL were similar in both plerixafor groups. However, in MM, the rescue strategy had more patients with stable or progressive disease, while the preemptive strategy had more patients with a complete or partial response.

**TABLE 1:** Demographic and clinical characteristics of the study participants.

Characteristic	Preemptive plerixafor *n* = 82	Rescue plerixafor *n* = 203	*p* *
Age, years			0.65**
	57 (46–63) 52.9 ± 13.1	57 (47–63) 53.7 ± 13.1	
Sex, male			0.794^a^
	38 (46.3%)	98 (48.3%)	
Race			0.735
White	48 (58.5%)	114 (56.2%)	
Black	5 (6.1%)	18 (8.9%)	
Mixed	29 (35.4%)	71 (35.0%)	
Diagnosis			0.517
MM	56 (68.3%)	140 (69.0%)	
NHL	11 (13.4%)	35 (17.2%)	
HL	15 (18.3%)	28 (13.8%)	
Staging			0.441
I	6 (7.3%)	10 (4.9%)	
II	26 (31.7%)	51 (25.1%)	
III	32 (39.0%)	87 (42.9%)	
IV	11 (13.4%)	38 (18.7%)	
No information	7 (8.5%)	17 (8.4%)	
Disease status			
MM			0.027
CR/VGPR	23 (41.1%)	56 (40.0%)	
SD	5 (8.9%)	25 (17.9%)	
PD	1 (1.8%)	10 (7.1%)	
No information	1 (1.8%)	10 (7.1%)	
Lymphomas			0.761
CR	10 (38.5%)	28 (44.4%)	
PR	13 (50.0%)	27 (42.9%)	
SD	1 (3.8%)	1 (1.6%)	
PD	2 (7.7%)	7 (11.1%)	
No information	0 (0%)	0 (0%)	
N° of treatment lines			0.67*
1	51 (62.2%)	115 (56.7%)	
2	20 (24.4%)	59 (29.1%)	
≥ 3	11 (13.4%)	29 (14.3%)	
Radiotherapy			0.161*
	18 (22.0%)	29 (14.3%)	
Previous ASCT			0.306^a^
	4 (4.9%)	17 (8.4%)	
N° chemotherapy cycles			0.564**
	7 (5–9)	7 (6–9)	
	7.7 ± 3.1	8.0 ± 4.2	

*Note:* Date presented as *n* (%), median (interquartile range), mean ± standard deviation.

Abbreviations: *n*, Number of patients; MM, Multiple Myeloma; HL, Hodgkin's Lymphoma; NHL, Non‐Hodgkin's Lymphoma; CR, Complete Response; VGPR, Very Good Partial Response; PR, Partial Response; SD, Stable Disease; PD, Progressive Disease; ASCT, Autologous Stem Cell Transplantation.

* Chi‐square test.

** Student's *t*‐test.

^a^ Fisher's exact test.

### 3.2 Clinical Outcomes

#### 3.2.1 Primary Clinical Outcomes

**FIGURE 1:**
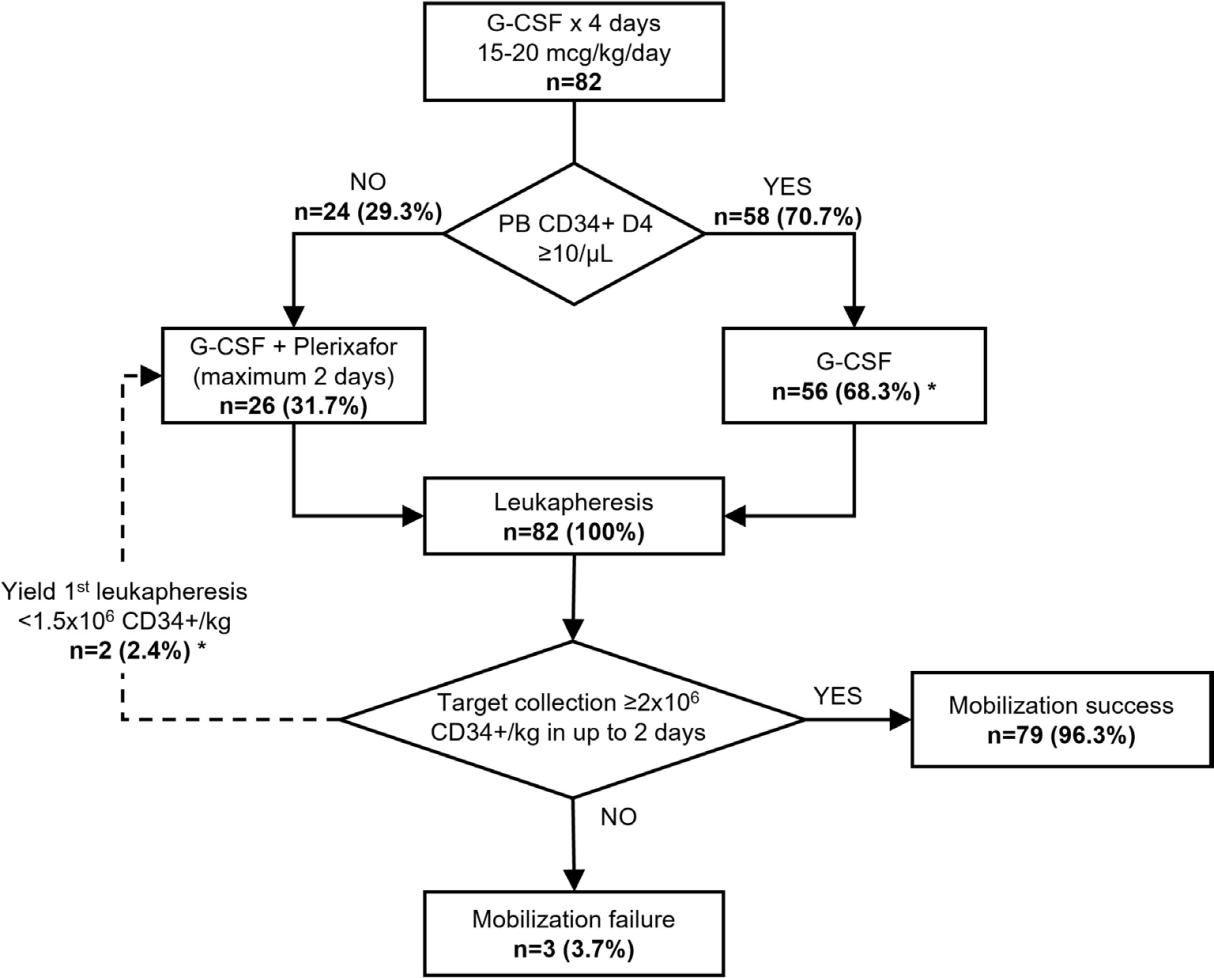
Flowchart of mobilization and collection of HSCs from the preemptive plerixafor strategy. *Two patients who had a CD34+ count in peripheral blood on D4 ≥ 10/μL had a yield of the first leukapheresis < 1.5 × 10^6^ CD34+/kg and received preemptive plerixafor. CD34+, Cluster of Differentiation 34 positive cells; D4, day 4; G‐CSF, Granulocyte Colony‐Stimulating Factor; HSC, hematopoietic stem cells; PB, peripheral blood.

**FIGURE 2:**
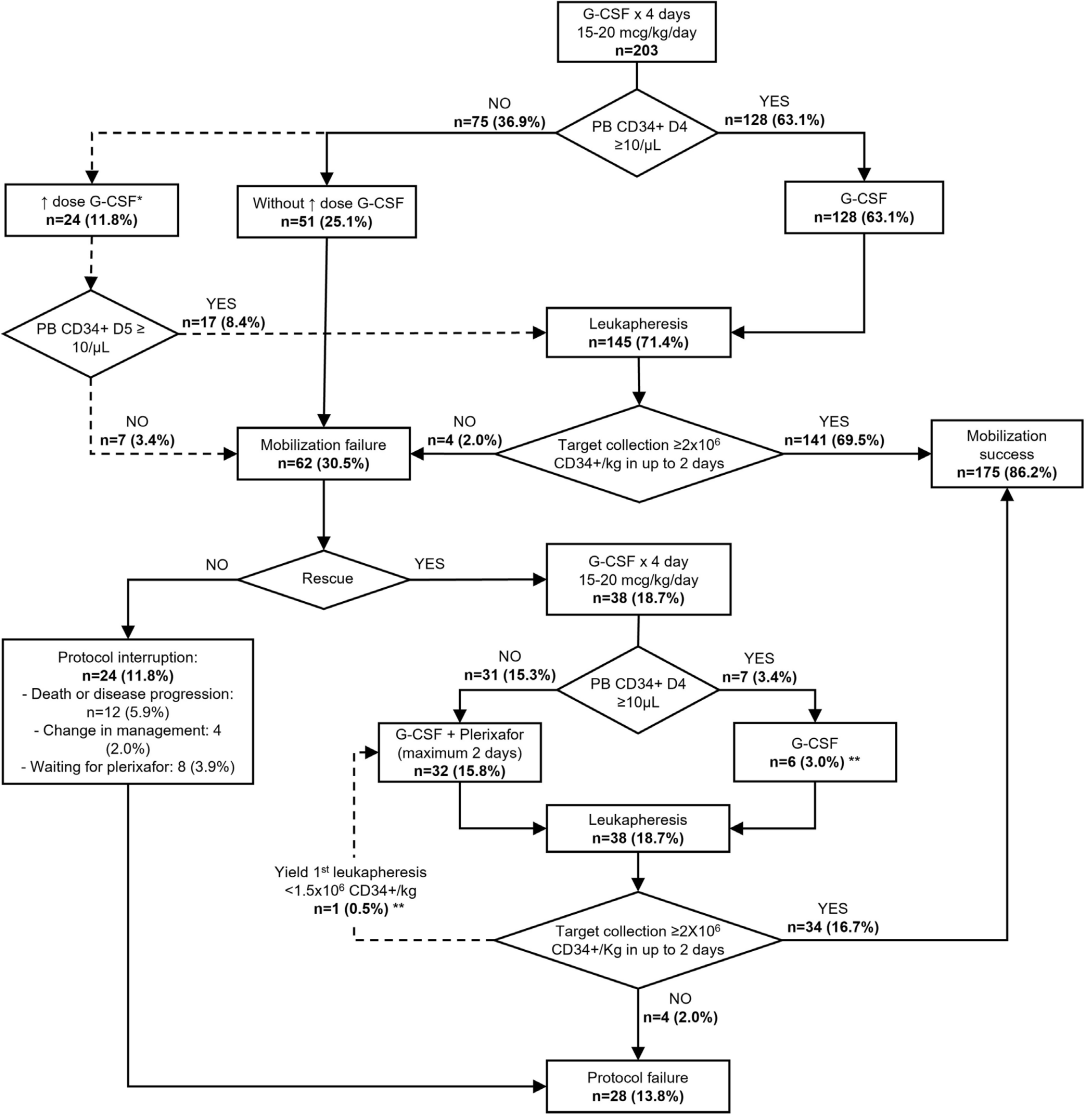
Flowchart of mobilization and collection of HSCs from the rescue plerixafor strategy. *As per protocol, at the medical team's discretion, 24 patients received an increase in G‐CSF dosage on D4 and D5, with a new CD34+ count on D5. **One patient who had a CD34+ count in peripheral blood on D4 ≥ 10/μL had a yield of the first leukapheresis < 1.5 × 10^6^ CD34+/kg and received preemptive plerixafor. CD34+, Cluster of Differentiation 34 positive cells; D4, day 4; D5, day 5; G‐CSF, Granulocyte Colony‐Stimulating Factor; HSC, hematopoietic stem cells; PB, peripheral blood.

Figures 1 and 2 present the flowchart of the mobilization and collection of HSCs from the preemptive plerixafor strategy and rescue plerixafor strategy, respectively. Table 2 summarizes the outcomes of PB stem cell mobilization and collection via leukapheresis, including remobilizations in both groups. The mobilization failure rate with G‐CSF was comparable in both strategies. Specifically, the rate of poor mobilization, defined as day 4 PB CD34+ count < 10/μL, was 29.3% in the preemptive group and 36.9% in the plerixafor rescue group, with no statistically significant difference. The preemptive group had more patients reach the minimum target of 2 × 10^6^ CD34+ cells/kg, and more patients proceeded to ASCT. Additionally, the preemptive group's interval between the start of first mobilization and ASCT was shorter. Both strategies showed no significant differences in the average total number of CD34+ cells collected and leukapheresis sessions.

**TABLE 2:** Mobilization and collection results by leukapheresis in study participants.

Variables	Preemptive plerixafor *n* = 82	Rescue plerixafor *n* = 203	*p* *
PB CD34+ cells count (D4 G‐CSF)	20 (8.25–33.86) 25.06 ± 23.74	13.1 (5.15–27) 24.85 ± 36.33	0.082
Poor mobilizer D4 (G‐CSF) 1st mobilization	24 (29.3%)	75 (36.9%)	0.272
Total CD34+ collected (×10^6^/kg)	3.70 (2.62–6.05) 4.78 ± 2.77	3.81 (2.77–6.23) 4.84 ± 2.96	0.896
Mobilization success (minimum collection ≥ 2 × 10^6^ CD34+ cells/kg)	79 (96.3%)	175 (86.2%)	0.01
Optimal collections	40 (48.8%)	88 (43.4%)	0.432
Remobilization	0	38 (18.7%)	< 0.001
Remobilization without plerixafor	0	6 (3%)	0.187
ASCT	71 (86.6%)	149 (73.4%)	0.019
Days between 1st mobilization and autologous ASCT	28 (8.5–108) 72.00 ± 77.26	90 (40–161) 128.43 ± 144.25	< 0.001
Days between 1st mobilization and remobilization	NA	97 (30–227.5) 134.5 ± 106.88	NA
N° Leukapheresis sessions	1 (1–1) 1.17 ± 0.38	1 (1–1) 1.07 ± 0.56	0.092
1	68 (82.9%)	148 (81.8%)	0.864
≥ 2	14 (17.1%)	33 (18.2%)	

*Note:* Data presented as *n* (%), median (interquartile range), mean ± standard deviation.

* Continuous variables: Mann–Whitney U test; categorical variables: Fisher's exact test; PBSC, Peripheral Blood Stem Cells; ASCT, Autologous Stem Cell Transplantation; D, day; CD, cluster of differentiation; NA, not applicable.

#### 3.2.2 Secondary Clinical Outcomes

In the ASCT data, no significant differences were noted between the two groups regarding the time required for platelet engraftment to exceed 50 000/mm^3^, which was shorter in the preemptive plerixafor group compared to the rescue plerixafor group (*p* = 0.017).

### 3.3 Leukapheresis

In both preemptive and rescue strategies, leukapheresis was performed using three different cell separation devices, with equivalent numbers using each device (*p* = 0.783). All patients in the preemptive plerixafor group underwent at least one leukapheresis session, with 13 (15.9%) patients requiring a second day for adequate stem cell collection. In the plerixafor rescue group, 145 (71.4%) underwent HSC collection by leukapheresis on the first mobilization with G‐CSF, and of these, 23 (15.9%) required a second day of leukapheresis. Of the 38 (18.7%) patients in the plerixafor rescue group who required remobilization, 10 (26.3%) required a second day of leukapheresis at remobilization.

In both strategies, there was no difference in the first leukapheresis session in the number of volumes processed in liters (4.1 ± 0.6 vs. 4.0 ± 0.6, *p* = 0.268) or processing time in minutes (2.95 ± 43 vs. 290 ± 35, *p* = 0.355). Similarly, in the second leukapheresis session, processed volumes (3.5 ± 1.1 vs. 3.4 ± 0.7, *p* = 0.817) and processing time (240 ± 64 vs. 254 ± 55, *p* = 0.552) were equivalent.

There was no statistically significant difference in the CEC between the two groups, with a mean of 0.16 ± 0.22 in the preemptive group versus a mean of 0.20 ± 0.32 in the rescue group (*p* = 0.377) (median, IQR 0.09, 0.06 to 0.18 vs. 0.11, 0.07 to 0.19, *p* = 0.118).

### 3.4 Costs

The preemptive strategy presented higher costs with the plerixafor medication and central venous catheter, while the rescue strategy involved costs with medical and nursing consultations and day hospital services. The average total cost per patient was higher in the preemptive plerixafor group (US$ 5642.54 ± 4516.40) compared to the rescue plerixafor group (US$ 4110.11 ± 3925.37), *p* = 0.006. The preemptive group utilized more plerixafor vials than the rescue group (*p* = 0.002). Cost estimates are detailed in Table 3 and Table S2.

**TABLE 3:** Cost estimates.

Costs	Preemptive plerixafor *n* = 82	Rescue plerixafor *n* = 203	*p* *
G‐CSF			0.429
	US$ 300.82 (247.50–376.03)	US$ 376.03 (240.66–451.24)	
	US$ 336.41 ± 87.40	US$ 359.62 ± 143.44	
Plerixafor			0.001
	US$ 0.00 (0.00–7.493.88)	US$ 0.00 (0.00–0.00)	
	US$ 2650.27 ± 4141.89	US$ 1273.59 ± 3309.07	
Laboratory tests			0.374
	US$ 179.82 (133.12–256.51)	US$ 243.12 (133.12–262.48)	
	US$ 199.11 ± 76.34	US$ 223.41 ± 108.83	
Medical consultations			< 0.001
	US$ 4.33 (4.33–4.33)	US$ 4.33 (4.33–4.33)	
	US$ 4.33 ± 0.00	US$ 5.15 ± 1.69	
Nursing consultations			< 0.001
	US$ 2.73 (2.73–2.73)	US$ 2.73 (2.73–2.73)	
	US$ 2.73 ± 0	US$ 3.37 ± 1.50	
Hospital day‐care			0.033
	US$ 59.73 (59.73–79.64)	US$ 59.73 (59.73–79.64)	
	US$ 65.02 ± 18.84	US$ 72.87 ± 27.72	
Inpatients care			0.150
	US$ 0.00 (0.00–34.68)	US$ 0.00 (0.00–0.00)	
	US$ 15.86 ± 24.25	US$ 14.20 ± 33.78	
CVC			< 0.001
	US$ 50.20 (50.20–50.20)	US$ 50.20 (0.00–50.20)	
	US$ 78.99 ± 62.60	US$ 31.95 ± 25.16	
Apheresis and cryopreservation**			0.632
	US$ 1968.46 (1968.46–1968.46)	US$ 1968.46 (1968.46–2003.14)	
	US$ 2289.53 ± 716.63	US$ 2103.13 ± 1070.91	
Total cost/patient			0.006
	US$ 2754.91 (2566.79–10117.92)	US$ 2659.26 (2495.96–4531.60)	
	US$ 5642.54 ± 4516.40	US$ 4110.11 ± 3925.37	

*Note:* Data presented as median (interquartile range), mean ± standard deviation.

* Mann–Whitney U test.

** Cost with apheresis, conditioning, and cryopreservation of HSCs, CD34+ peripheral blood count, CD34+/leukapheresis product count, and transfusions during apheresis collection; G‐CSF, granulocyte colony‐stimulating factor; CVC, central venous catheter; US$, US Dollars. Values in Brazilian Reais from 2024 converted to US Dollars in the same year using the CCEMG—EPPI‐Centre Cost Converter (v.1.6)—https://eppi.ioe.ac.uk/costconversion/.

### 3.5 Incremental Cost‐Effectiveness Ratio

Table 4 summarizes the ICER results for various outcomes comparing preemptive and rescue plerixafor strategies. These ICER calculations incorporated an incremental cost difference of +US$ 1532.44. The estimated incremental effectiveness was 10.1% for achieving successful minimum collection, 13.2% for an additional percentage of patients proceeding to ASCT, and 4.7% for patients achieving optimal collection. When considering a mean difference of 0.1 in the number of leukapheresis sessions, the preemptive plerixafor protocol resulted in higher costs and more days of leukapheresis.

**TABLE 4:** Incremental cost‐effectiveness ratio (ICER) for base case analysis.

Outcomes	Preemptive plerixafor	Rescue plerixafor	Difference (∆)	ICER (∆ cost/ ∆ outcome)
Total cost (mean)	US$ 5642.35	US$ 4110.11	US$ 1532.44	—
% collection ≥ 2 × 10^6^ CD34+ cells/kg (minimum collection)	96.34	86.21	10.13	US$ 151.28
% patients who progressed to ASCT	86.59	73.40	13.19	US$ 116.18
% collection ≥ 4 × 10^6^ CD34+ cells/kg (optimal collection)	47.56	42.86	4.70	US$ 326.05
Leukapheresis sessions (mean)	1.17	1.07	0.1	−US$ 15324.36 (dominated)*

*Note:* Values in Brazilian Reais from 2024 converted to US Dollars in the same year using the CCEMG–EPPI‐Centre Cost Converter (v.1.6)—https://eppi.ioe.ac.uk/costconversion/.

Abbreviations: ASCT, autologous stem cell transplantation; ∆, the difference between preemptive and rescue group; CD, Cluster of differentiation; US$, US Dollars.

* ICER per avoided leukapheresis session. ICER, incremental cost‐effectiveness ratio.

#### 3.5.1 Analysis of ICER Uncertainties

##### 3.5.1.1 Deterministic Analyses Sensitivity

The tornado charts of the sensitivity analyses for each clinical outcome are presented in Figure S4. For clinical outcomes such as the percentage of successful minimum collections and the percentage of progression to ASCT, variations in ICER parameters did not show significant variation, indicating a lower degree of uncertainty in the base case ICER results. The parameter that caused the most variation in ICER values was the lower limit of the 95% CI for preemptive plerixafor. The greatest degree of uncertainty was in the clinical outcome of optimal collections, where parameter variations led the ICER to dominate results.

Tables S3–S5 respectively present the deterministic sensitivity analyses with variation in the average cost per patient in each strategy (95% CI), variation in the price of the drug G‐CSF (filgrastim), and variation in the mean of each clinical outcome (95% CI).

##### 3.5.1.2 Probabilistic Analysis Sensitivity

Table S6 provides mean ICERs and their 95% CI derived from bootstrapping, using 10 000 random samples with replacement for the four clinical outcomes. The average ICERs closely align with the base case values.

**FIGURE 3:**
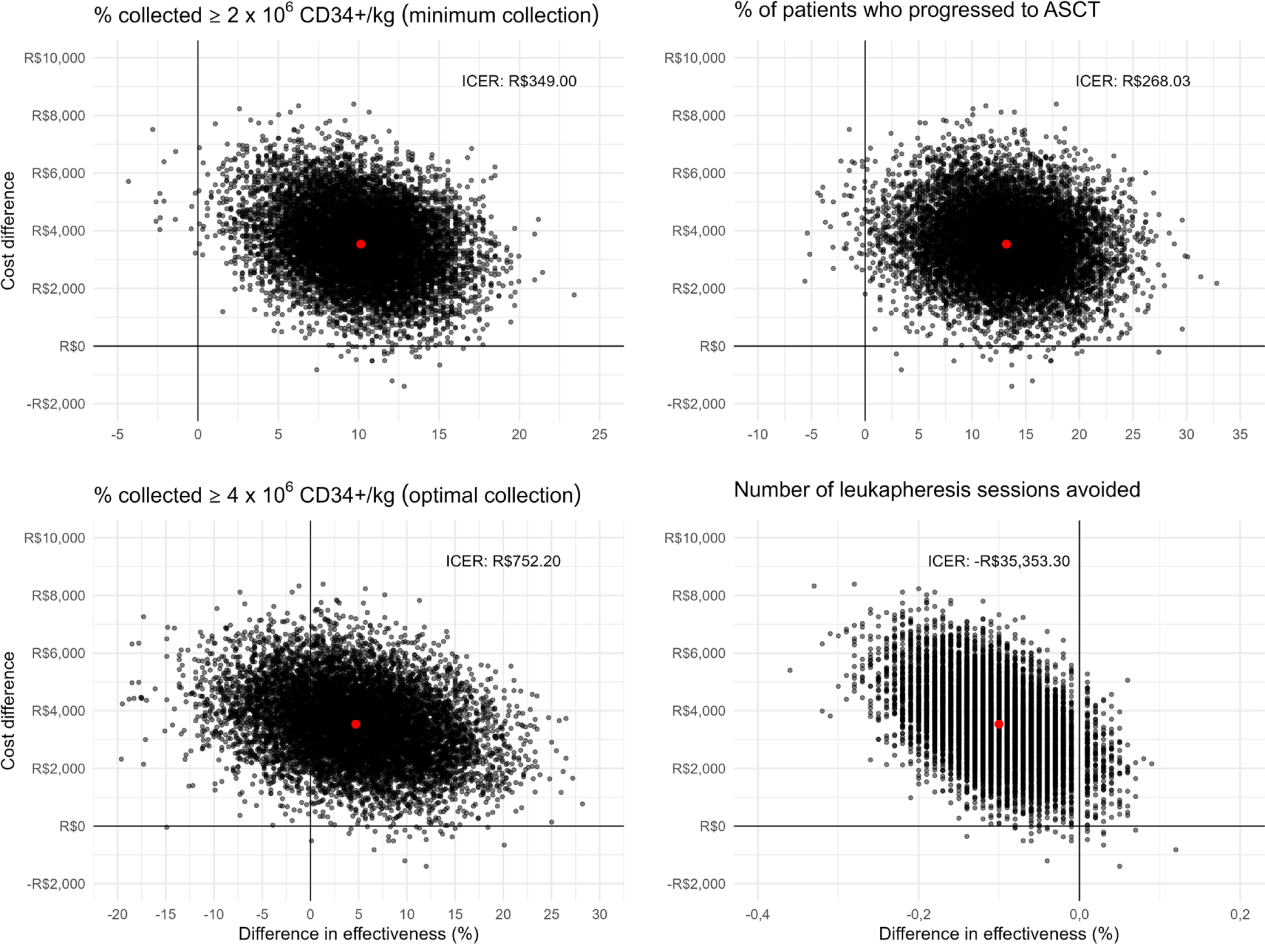
Scatterplot of the bootstrap cost‐effectiveness estimates. 99.6% of the bootstrap resampling remained in the base case quadrant for minimum collections; 99.3% for ASCT progression, and 97.04% for apheresis. For optimal collections, 23.3% shifted to the dominated quadrant and 76.4% remained in the base case quadrant. ASCT, autologous stem cell transplantation; CD34+, Cluster of Differentiation 34 positive cells.

In the Cost‐Effectiveness Plan (Figure 3), we observed that for the three outcomes (% minimum collections, % progression to ASCT, and number of leukapheresis sessions), more than 97% of the bootstrapped ICERs (black dots) remained in the same quadrant as the base case, represented by the red dot. However, for % optimal collections, 23.3% of the bootstrapped ICERs were dominated, indicating a higher degree of uncertainty in the base case for this outcome.

**FIGURE 4:**
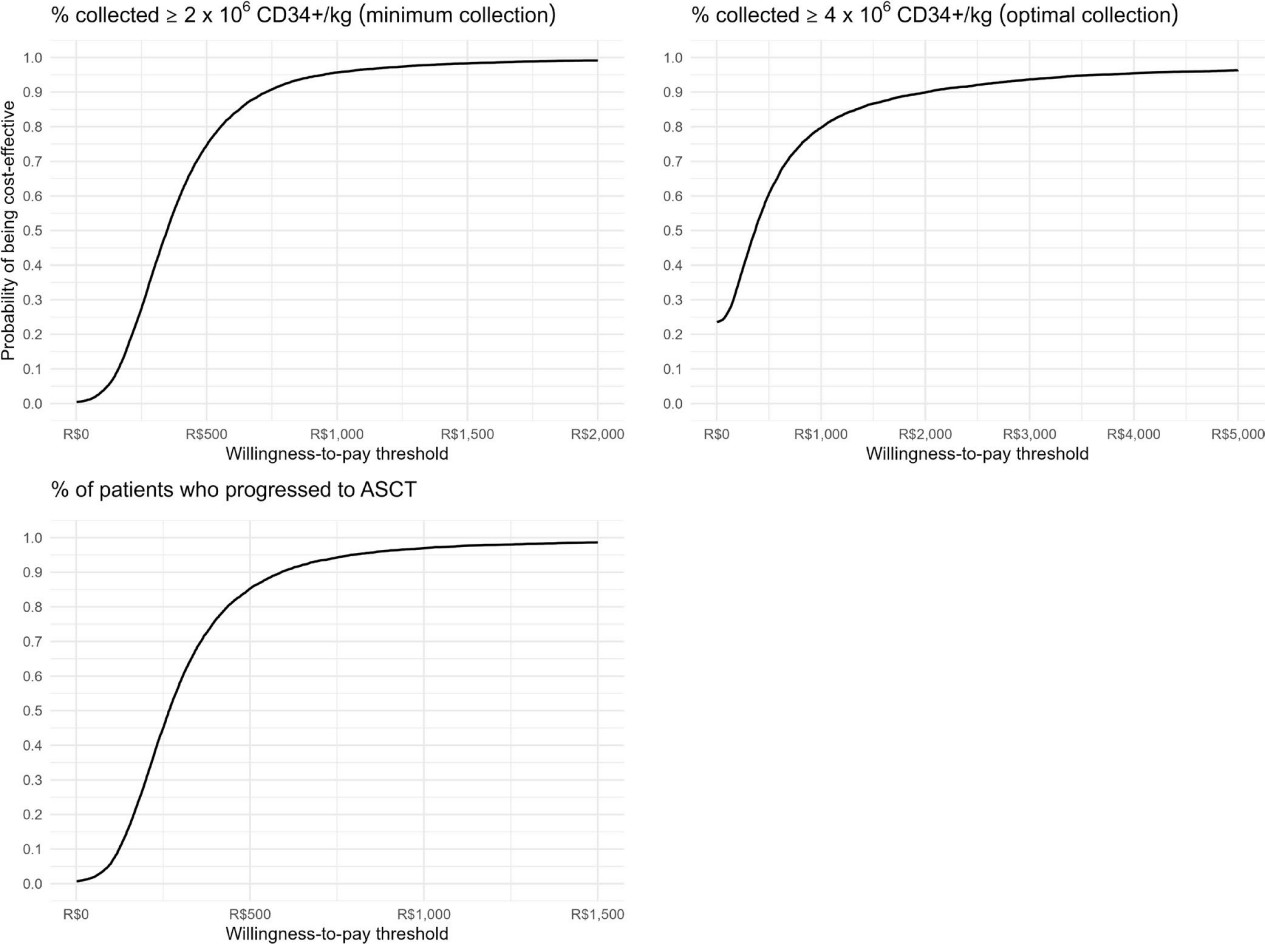
Cost‐effectiveness acceptability curves. ASCT, autologous stem cell transplantation; CD34+, Cluster of Differentiation 34 positive cells.

Cost‐effectiveness acceptability curves were generated for each clinical outcome. With a willingness‐to‐pay threshold of US$ 433.46, 95.7% of the assumptions were cost‐effective for successful minimum collections, 96.9% for progression to ASCT, and 79.7% for optimal collections, respectively (Figure 4).

### 3.6 Subgroup Analysis

#### 3.6.1 Subgroup by Diagnosis

Table S7 reports patients' characteristics and outcomes according to their diagnosis: MM or lymphoma (HL or NHL).

Among patients diagnosed with MM, there was no difference between the two mobilization strategies in terms of efficacy. However, in the preemptive strategy, the interval between the first mobilization and the ASCT was shorter (mean 64.47 ± 73.09 vs. 112.39 ± 126.67, *p* = 0.024). In the preemptive strategy, no remobilization was required, whereas in the rescue strategy, 15.7% of patients underwent remobilization. The average cost was higher in the preemptive strategy, with statistical significance.

In the lymphoma group, the rescue strategy showed a higher remobilization rate, while the interval between the first mobilization and ASCT was shorter in the preemptive strategy (mean 76.62 ± 78.81 vs. 149.94 ± 149.53, *p* = 0.042). Additionally, there was no difference in the total average cost per patient between the two strategies.

The calculations of ICERs for each clinical outcome were performed separately for MM and lymphomas, and the results can be found in Tables S8 and S9, respectively. In both groups, the ICER results were like the total group. The ICER for the number of leukapheresis sessions remained dominated, and for the other three outcomes, the ICER remained below US$ 1000.

#### 3.6.2 Subgroup of Poor Mobilizers

Patients' characteristics, clinical outcomes, and cost estimates for poor mobilizers, overall and by diagnosis are presented in Table S10.

Considering only poor mobilizers, the analysis resulted in larger incremental costs for preemptive plerixafor versus rescue plerixafor. However, incremental effectiveness was also greater in the overall sample, resulting in ICER values like the base case (Table S11). The larger difference in costs was also observed in the analysis by diagnosis, in both MM and lymphoma patients.

Since clinical outcomes remained similar to those observed in the total MM group, the ICER increased for progression to ASCT and optimal collection, though remaining under US$ 2000 (Table S12). Higher incremental effectiveness was also observed in the lymphoma subgroup, attributable to worse clinical outcomes in the rescue plerixafor group, further widening the gap between interventions, resulting in ICER values close to those observed for the complete subgroup of lymphoma patients (Table S13).

## 4 Discussion

The preemptive plerixafor approach, although associated with higher upfront costs due to the immediate availability of the drug, demonstrated superior clinical outcomes compared to the rescue plerixafor strategy. There was a lower mobilization failure rate, reduced requirement for remobilization, expedited transition to ASCT, and a higher proportion of patients successfully progressing to ASCT. This resulted in a low ICER for most of the clinical outcomes analyzed.

The preemptive plerixafor strategy was implemented selectively for cases of mobilization failure despite administration of G‐CSF or chemotherapy + G‐CSF, based on PB CD34+ cell counts on D 4 of mobilization or pre‐leukapheresis [11]. This approach necessitated immediate availability of the medication in the event of failure, resulting in substantial financial implications [18, 19, 20, 21, 22, 23, 24]. In the rescue strategy, plerixafor was utilized for remobilization following initial mobilization failure despite treatment with G‐CSF alone or in combination with chemotherapy [13]. Compassionate use studies have reported success rates ranging from approximately 64% to 90%, with 56% to 84% of patients proceeding to ASCT [18, 19, 20, 21, 22, 23]. However, delaying stem cell remobilization may necessitate additional chemotherapy cycles, which can increase the risk of disease relapse and other complications, in addition to escalating costs [13].

Utilizing a modified rescue plerixafor strategy contingent upon CD34+ cell counts following the failure of G‐CSF, almost 90% of our patients achieved successful mobilization with the rescue plerixafor strategy, with more than 70% proceeding to ASCT. This was consistent with reported findings from other studies [25, 26, 27, 28, 29, 30].

The goal of the modified rescue plerixafor strategy is to optimize plerixafor usage in patients remobilized with isolated G‐CSF. The literature indicates an 81.6% failure rate with G‐CSF alone for remobilization [31, 32]. Our findings are consistent with this, showing that 84.2% of patients did not meet the stem cell collection criteria with isolated G‐CSF, highlighting the necessity of plerixafor intervention. The preemptive plerixafor strategy demonstrated advantages over the rescue plerixafor strategy, including a shorter time to ASCT initiation and a higher transplantation rate. Vishnu et al. reported that 95% of upfront plerixafor recipients underwent ASCT, compared to 75% of patients who received G‐CSF alone [33]. Similarly, Micallef et al. found higher ASCT rates with preemptive plerixafor (98%) compared to G‐CSF alone (76%) [34]. Additionally, the time to platelet and leukocyte engraftment post‐ASCT did not differ between plerixafor strategies [34, 35, 36, 37].

It is noteworthy that previous studies compared plerixafor with isolated G‐CSF [33, 34, 36]. Different plerixafor protocols [37] have demonstrated a reduction in leukapheresis sessions, a benefit not observed in our comparison. Organizational aspects of our investigational site may have influenced this outcome, as our institutional protocol limits leukapheresis to two days per patient per mobilization. Consequently, if a patient required three or more sessions for collection, this would not be observed in our study. While no benefit was observed in reducing leukapheresis sessions, all preemptive plerixafor patients met leukapheresis criteria, unlike 28.6% of patients in the rescue plerixafor strategy. Reserved leukapheresis equipment during mobilization remains underutilized if mobilization fails, incurring costs for hemotherapy services that could be allocated elsewhere [38].

Plerixafor based approaches, despite its advantages, are associated with high costs [35]. In our study, the average cost per patient was higher in the preemptive plerixafor strategy compared to the rescue plerixafor strategy (*p* < 0.001), primarily due to higher plerixafor drug costs. However, costs for laboratory tests, medical consultations, nursing visits, and day hospital services were significantly higher in the rescue plerixafor group. Economic evaluations generally consider quality of life (QALY), or life years gained as effectiveness outcomes [39]. Although QALY and life years gained were not used as outcomes in this study, the values obtained for alternative outcomes were well below the cost‐effectiveness thresholds declared by the National Committee for Health Technology Incorporation (Comissão Nacional de Incorporação de Tecnologias; CONITEC) in August 2022 for QALY and years of life gained, respectively [40]. Additionally, through bootstrap analysis, the cost‐effectiveness acceptability curve indicated that, with an incremental cost of US$ 433.46, over 95% of the simulations conducted were considered cost‐effective for both primary clinical outcomes. Therefore, preemptive plerixafor could be considered potentially cost‐effective for Brazil.

The literature includes several economic evaluations comparing plerixafor with G‐CSF, but a few studies assess the two plerixafor strategies analyzed in our study. A cost‐effectiveness evaluation conducted in the Czech Republic compared the two strategies, showing lower costs and greater clinical effectiveness for preemptive plerixafor compared to rescue, making the preemptive strategy cost‐effective. However, the study design included only poor mobilizers, excluding good mobilizers [15]. Our results for the subgroup of poor mobilizers did not differ from the overall sample analysis. Although the incremental cost was larger in this subgroup analysis, the effectiveness gap was also widened, likely due to the absence of remobilization in some patients, which impacted both costs and clinical results in the rescue plerixafor group. However, we believe that focusing exclusively on poor mobilizers may introduce bias, as both good and poor mobilizers are part of the overall strategy. Including all eligible patients provides a more comprehensive and realistic view of the intervention's impact. The higher cost of plerixafor in the preemptive strategy could also be due to delays in plerixafor procurement in the rescue strategy. The average delay in our study was 129 ± 105 days. During this time, some patients in the rescue group experienced disease progression, death, or opted out of ASCT (11.8%). As a result, fewer patients underwent remobilization in the rescue plerixafor group, potentially contributing to the lower medication cost in this strategy.

The deterministic sensitivity analysis showed that the base case ICERs were more sensitive to variation in the clinical outcomes of the preemptive plerixafor strategy. Therefore, to ensure the cost‐effectiveness of preemptive plerixafor, it is necessary to guarantee good clinical outcomes, such as achieving the minimum stem cell collection and providing a higher number of patients progressing to ASCT. Hence, evaluating the predictors of poor mobilization during stem cell mobilization consultations is important to guide strategies with better clinical and economic outcomes. The “Trapianto di Midollo Osseo” group developed a definition of “poor mobilizers” that is applicable in both clinical trials and clinical practice. They used the analytic hierarchy process to prioritize criteria for identifying these patients. This method helps to standardize the identification of individuals who may have difficulties in stem cell mobilization, contributing to more effective treatment strategies [41].

Limitations of this study include its retrospective design and intermittent unavailability of plerixafor, leading to delays in data collection for the preemptive group. The employment of clinical outcomes instead of outcomes such as QALYs and Life Years gained makes it difficult to compare results with other economic evaluation studies. The COVID‐19 pandemic further hindered patient remobilization, potentially biasing the rescue plerixafor group. Additionally, the protocol's restriction to two leukapheresis sessions per mobilization may have limited comprehensive data acquisition. Chemo‐ mobilization was excluded due to institutional protocol constraints. The use of three different brands of leukapheresis equipment for stem cell collection may have introduced measurement bias. However, a comparison of their collection results did not reveal significant differences. Retrospective studies comparing Optia and COBE apheresis systems demonstrate their comparability in stem cell collection efficiency. Notably, Optia exhibits higher efficiency with shorter procedures, lower cellular contamination, and consequently lower hematocrit and leukocyte counts. Due to logistical reasons, we do not perform CD34+ counts immediately before leukapheresis. The CEC calculation was based on the CD34+ count from PB on the 4th day of mobilization. Therefore, the CEC value may not accurately reflect the patient's actual condition immediately before apheresis.

Despite these limitations, this study provides the first economic evaluation of plerixafor's application in stem cell mobilization and collection in Brazil, based on primary data and real‐world experience. Given the imprudence of generalizing results across countries with diverse income levels, conducting such evaluations in varied contexts is crucial for informed decision‐making. Our analysis should guide further economic assessments in different settings, facilitating the more effective utilization of plerixafor in stem cell mobilization and collection for ASCT. As perspectives for future studies, it is essential to incorporate economic models that allow for longer‐term follow‐up, providing a more comprehensive view of the benefits and costs over time. Additionally, conducting analyses considering QALY or years of life gained can provide a more thorough assessment of the treatment's impact. Presenting this evidence to CONITEC is a crucial strategy to ensure the inclusion of plerixafor in the Brazilian SUS. To achieve this, it will be necessary to present a robust argument demonstrating not only the clinical efficacy of the medication but also its economic viability and its positive impact on patient's quality of life.

## 5 Conclusions

In the Brazilian SUS perspective, preemptive plerixafor is a cost‐effective strategy compared to rescue plerixafor for the mobilization of HSC in candidates for ASCT with MM and lymphoma. It offers higher efficacy with lower ICER values, making it a clinically beneficial option despite its higher cost.

## Author Contributions

R.M.A.P., C.A.N., and A.G.C. designed the study. R.M.A.P. and J.A.P. collected data from the medical records. R.M.A.P. and M.A.Z.M. wrote the manuscript and designed the figures. M.A.Z.M. and V.F.C. performed the data analysis. R.M.A.P. and J.A.P. translated the article, tables, and figures into English. All authors reviewed and approved the final version of this work.

## Ethics Statement

The study was approved by the Ethics Committees of the Hospital de Transplantes Euryclides de Jesus Zerbini/Hospital das Clínicas da Universidade de São Paulo (CAAE 12240319.7.0000.0091).

## Consent

Informed consent was obtained from prospective participants but was waived for the retrospective group.

## Conflicts of Interest

The authors declare no conflicts of interest.

## Supporting information


**Figure S1.** Preemptive plerixafor strategy.
**Figure S2.** Rescue plerixafor strategy.
**Figure S3.** Flow‐diagram of patient selection.
**Figure S4.** Tornado chart for each clinical outcome.

**Table S1.** Unit value table per resource for estimating cost per patient.
**Table S2.** Resource consumption for cost estimation.
**Table S3.** Sensitivity analysis by varying the average cost per patient in each strategy.
**Table S4.** Sensitivity analysis by varying the price of filgrastim.
**Table S5.** Sensitivity analysis by variation in clinical outcomes.
**Table S6.** Estimation of the mean incremental cost‐effectiveness ratio and confidence interval by bootstrapping.
**Table S7.** Demographic, clinical characteristics, clinical outcomes and cost estimates per disease.
**Table S8.** Incremental cost‐effectiveness ratios for multiple myeloma.
**Table S9.** Incremental cost‐effectiveness ratios for lymphomas (hodgkin and non‐hodgkin).
**Table S10.** Demographic, clinical characteristics, clinical outcomes, and cost estimates for poor mobilizers, overall and per disease.
**Table S11.** Incremental cost‐effectiveness ratios for poor mobilizers—overall.
**Table S12.** Incremental cost‐effectiveness ratios for poor mobilizers—multiple myeloma.
**Table S13.** Incremental cost‐effectiveness ratios for poor mobilizers—lymphomas (hodgkin and non‐hodgkin).

## Data Availability

The data that support the findings of this study are available from the corresponding author upon reasonable request.
