# Supplementary material for: Cost‐Effectiveness of Preemptive Plerixafor Versus Rescue Plerixafor for Mobilization and Collection of Hematopoietic Stem Cells in Patients With Multiple Myeloma and Lymphoma
Source: J Clin Apher. 2025 May 3;40(3):e70026. doi: 10.1002/jca.70026 (PMC12049149; doi:10.1002/jca.70026)
Supplement: Supplementary file 1 — Figure S1. Preemptive plerixafor strategy. Figure S2. Rescue plerixafor strategy. Figure S3. Flow‐diagram of patient selection. Figure S4. Tornado chart for each clinical outcome. [file JCA-40-e70026-s002.docx]

# SUPPLEMENTARY FIGURES

**Supplementary Figure 1 –** Preemptive plerixafor strategy

**
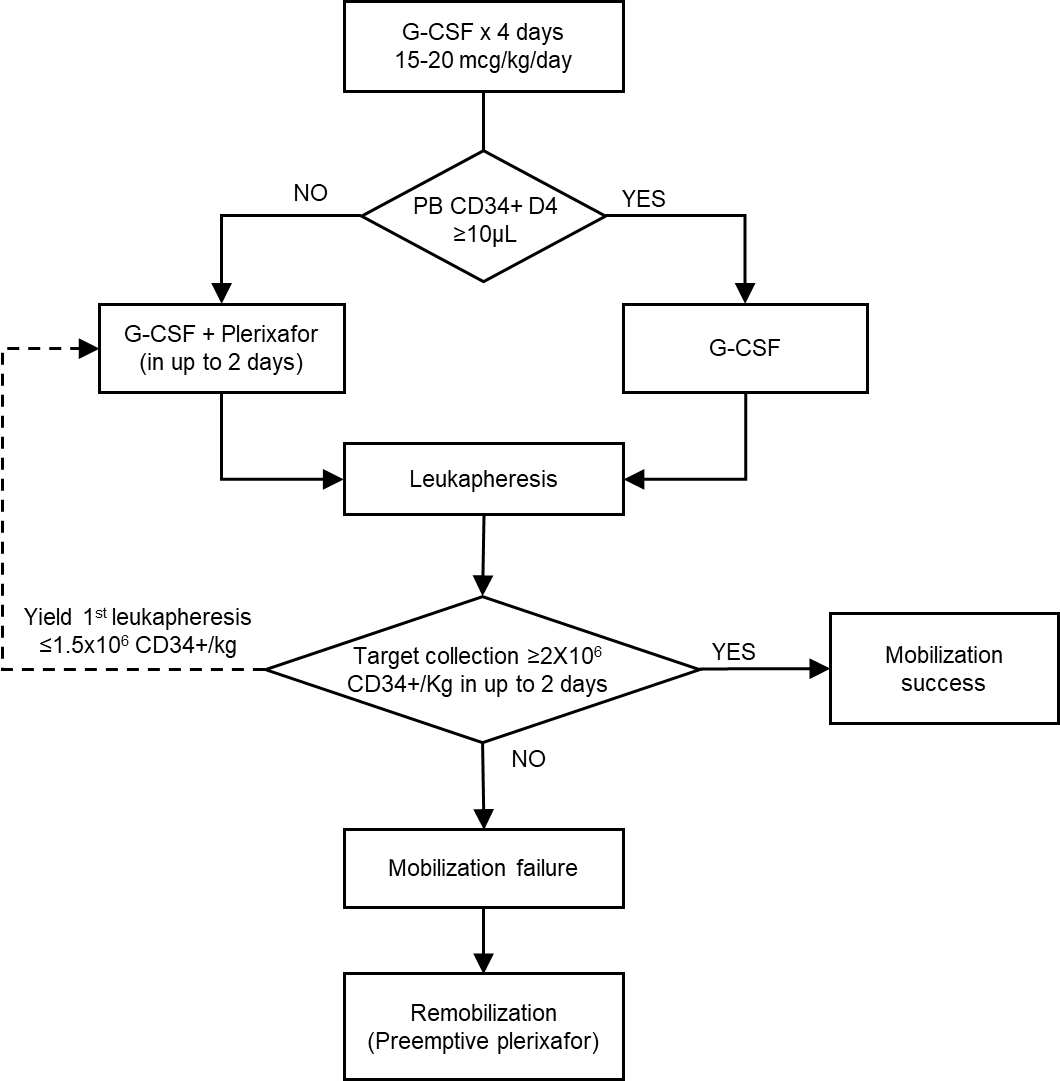
**

Legend: CD34+, Cluster of Differentiation 34 positive cells; D4, day 4; G-CSF, Granulocyte Colony-Stimulating Factor; PB, peripheral blood.

**Supplementary Figure 2 –** Rescue plerixafor strategy

**
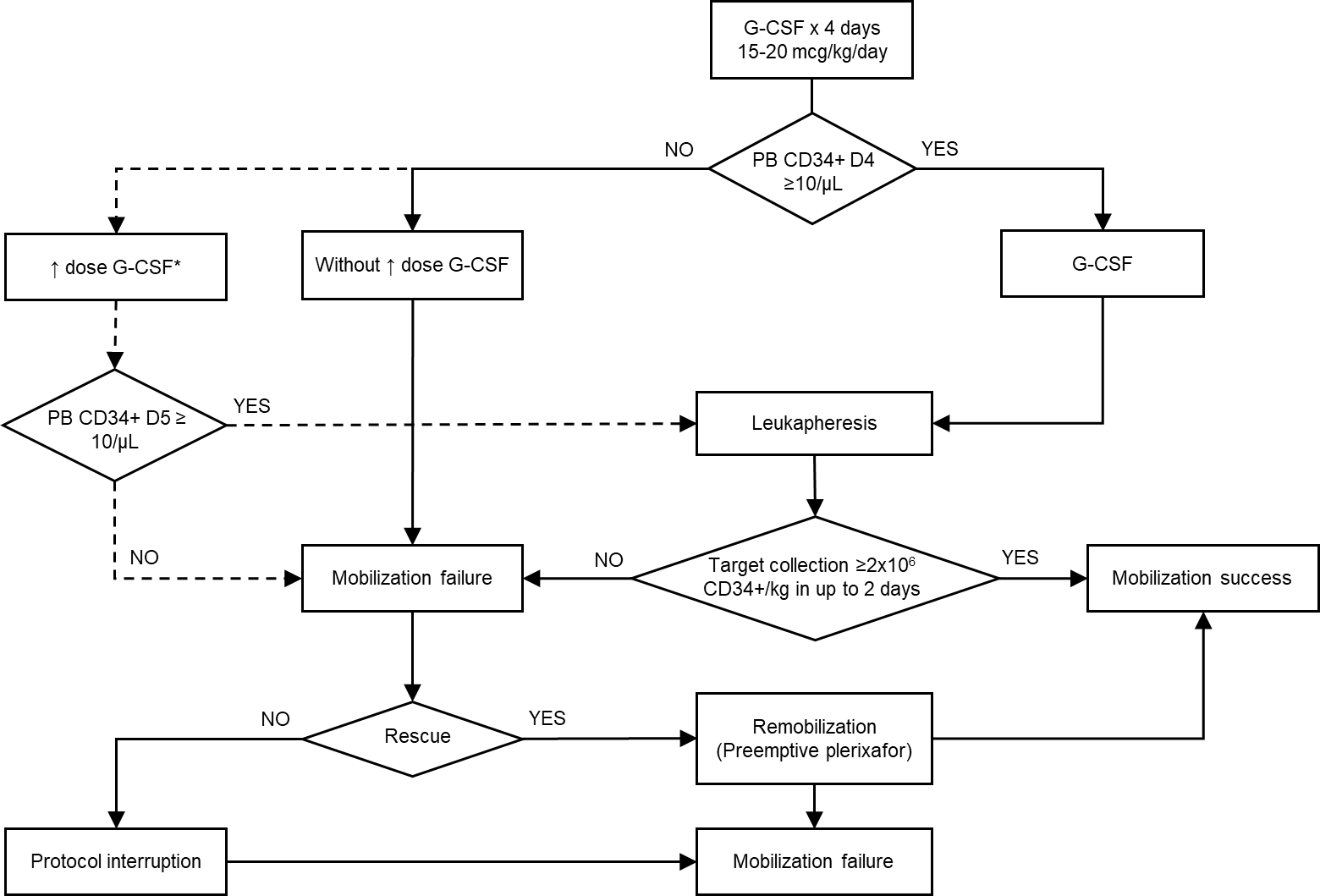
**

Legend: CD34+, Cluster of Differentiation 34 positive cells; D4, day 4; G-CSF, Granulocyte Colony-Stimulating Factor; PB, peripheral blood.

**Supplementary Figure 3 –** Flow-diagram of patient selection


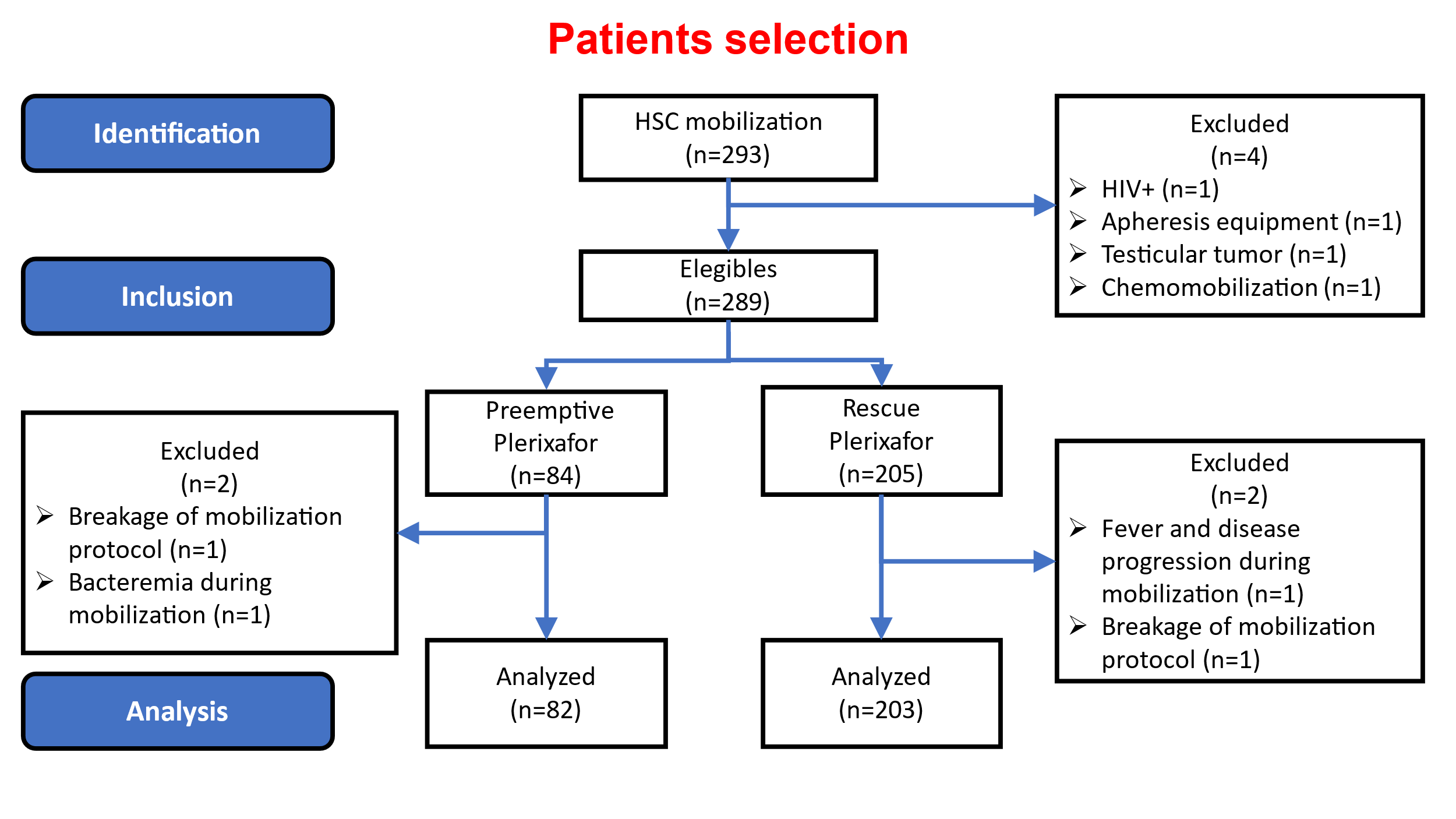


Legend: HSC, Hematopoietic Stem Cell; HIV, Human Immunodeficiency Virus.

**Supplementary Figure 4 –** Tornado chart for each clinical outcome

**
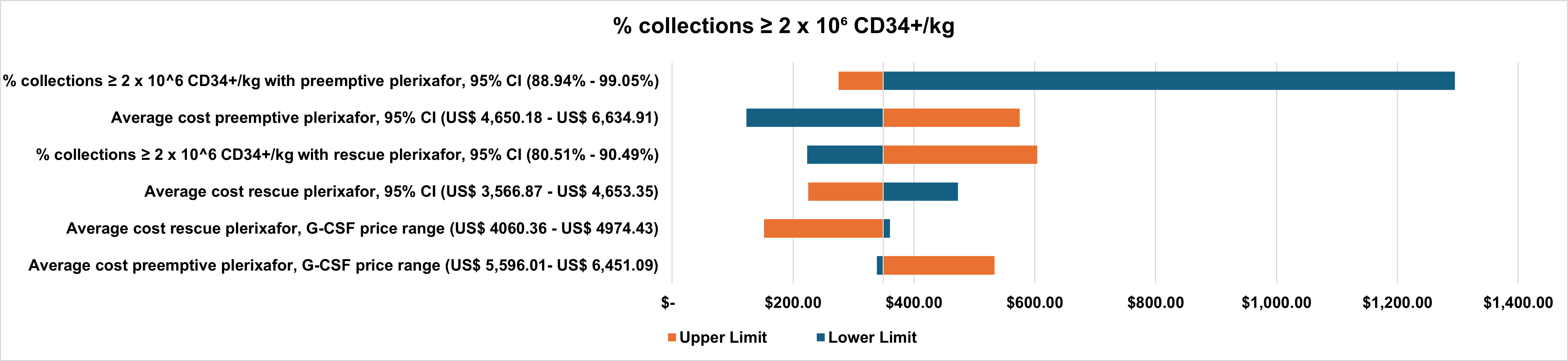
**

**
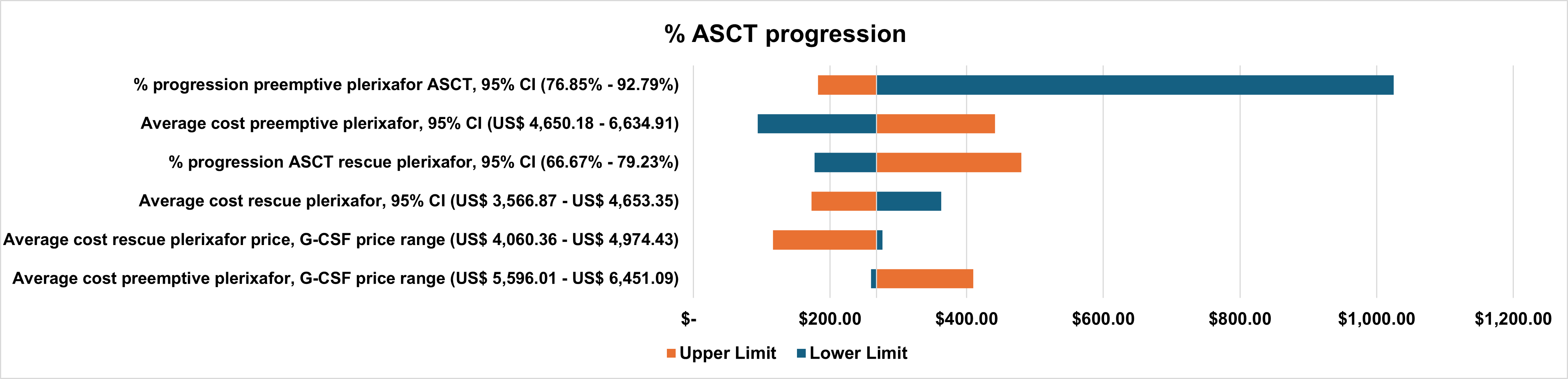
**

**
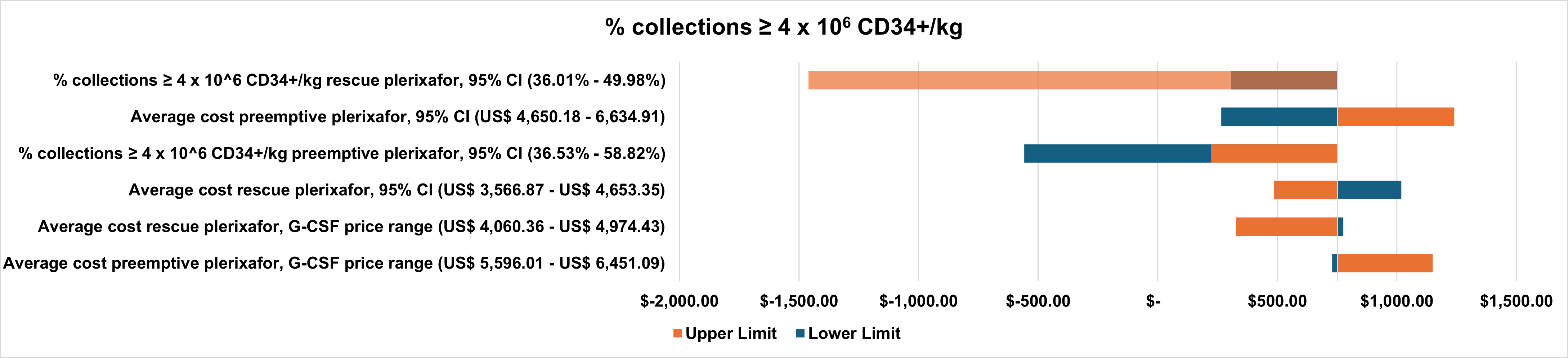
**

**
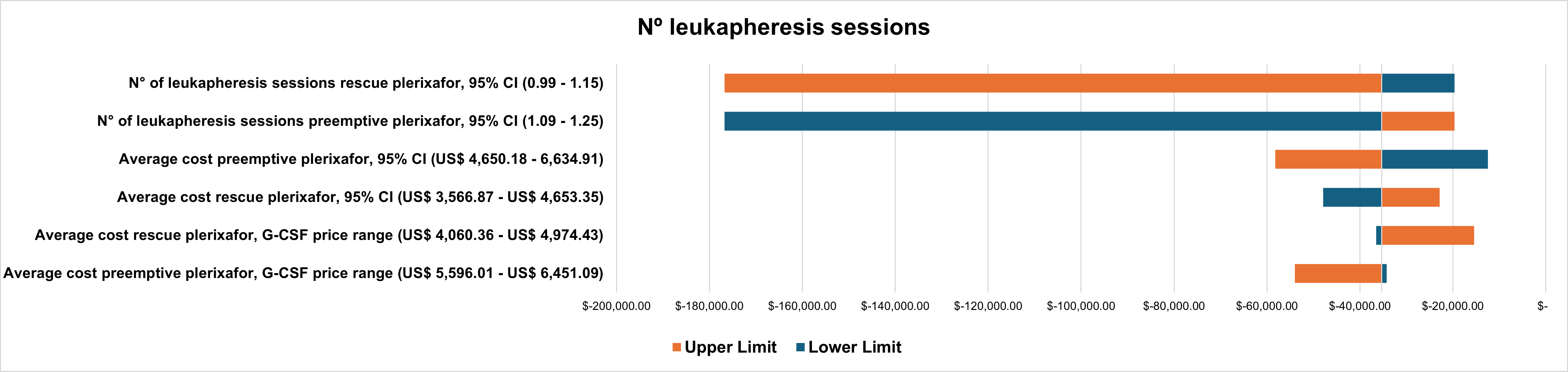
**

Legend: ASCT, autologous stem cell transplantation; CD34+, Cluster of Differentiation 34 positive cells; CI, confidence interval; G-CSF, Granulocyte Colony-Stimulating Factor; PB, peripheral blood
