# Supplementary material for: Cost‐Effectiveness of Preemptive Plerixafor Versus Rescue Plerixafor for Mobilization and Collection of Hematopoietic Stem Cells in Patients With Multiple Myeloma and Lymphoma
Source: J Clin Apher. 2025 May 3;40(3):e70026. doi: 10.1002/jca.70026 (PMC12049149; doi:10.1002/jca.70026)
Supplement: Supplementary file 2 — Table S1. Unit value table per resource for estimating cost per patient. Table S2. Resource consumption for cost estimation. Table S3. Sensitivity analysis by varying the average cost per patient in each strategy. Table S4. Sensitivity analysis by varying the price of filgrastim. Table S5. Sensitivity analysis by variation in clinical outcomes. Table S6. Estimation of the mean incremental cost‐effectiveness ratio and confidence interval by bootstrapping. Table S7. Demographic, clinical characteristics, clinical outcomes and cost estimates per disease. Table S8. Incremental cost‐effectiveness ratios for multiple myeloma. Table S9. Incremental cost‐effectiveness ratios for lymphomas (hodgkin and non‐hodgkin). Table S10. Demographic, clinical characteristics, clinical outcomes, and cost estimates for poor mobilizers, overall and per disease. Table S11. Incremental cost‐effectiveness ratios for poor mobilizers—overall. Table S12. Incremental cost‐effectiveness ratios for poor mobilizers—multiple myeloma. Table S13. Incremental cost‐effectiveness ratios for poor mobilizers—lymphomas (hodgkin and non‐hodgkin). [file JCA-40-e70026-s001.docx]

SUPPLEMENTARY TABLES

**Supplementary Table 1** – Unit Value Table per Resource for Estimating Cost per Patient

| **Procedure Codes** | **Resource** | **Unit price**  **(US$)** | **Source** |
| --- | --- | --- | --- |
| 02.02.02.038-0 | Complete blood count | 1.78 | SIGTAP |
| 02.02.01.060-0 | Serum potassium dosage | 0.80 | SIGTAP |
| 02.02.01.056-2 | Serum magnesium dosage | 0.87 | SIGTAP |
| 02.02.01.022-8 | Serum ionic calcium dosage | 1.52 | SIGTAP |
| 02.02.01.031-7 | Serum creatinine dosage | 0.80 | SIGTAP |
| 02.02.06.021-7 | Human chorionic  gonadotropin (hCG Test) | 3.40 | SIGTAP |
| 02.02.08.015-3 | Blood Culture | 4.98 | SIGTAP |
| 02.02.02.014-7 | Prothrombin Time and  Activity Determination | 1.18 | SIGTAP |
| 02.02.02.013-4 | Activated Partial Thromboplastin  Time Determination | 2.50 | SIGTAP |
| 02.02.03.064-4 | Anti-HBe antibody test | 8.04 | SIGTAP |
| 02.02.03.063-6 | Anti-HBs antibody test | 8.04 | SIGTAP |
| 02.02.03.078-4 | Total anti-HBc antibody test (IgG  and IgM) | 8.04 | SIGTAP |
| 02.02.03.097-0 | HBsAg test (Hepatitis B  Surface Antigen) | 8.04 | SIGTAP |
| 02.02.03.098-9 | HBeAg test (Hepatitis B  Envelope Antigen) | 8.04 | SIGTAP |
| 02.02.03.089-0 | IgM against Hepatitis B core  antigen (ANTI-HBC-IGM) | 8.04 | SIGTAP |
| 02.13.01.020-8 | Quantitative PCR for Hepatitis B | 73.03 | SIGTAP |
| 02.02.03.067-9 | Antibodies against Hepatitis C  virus (ANTI-HCV) | 8.04 | SIGTAP |
| 02.02.03.108-0 | Quantitative PCR for Hepatitis C | 73.03 | SIGTAP |
| 02.02.03.030-0 | Antibodies against HIV 1 and 2 (ELISA) | 3.49 | SIGTAP |
| 02.02.03.029-6 | Anti-HIV 1 (Western Blot) | 36.84 | SIGTAP |
| 02.02.03.004-0 | Detection of HIV-1 nucleic acids | 28.18 | SIGTAP |
| 02.02.03.031-8 | Antibodies against HTLV-1 and HTLV-2 | 8.04 | SIGTAP |
| 02.02.03.125-0 | HTLV1 virus RNA detection | 28.18 | SIGTAP |
| 02.02.03.085-7; 02.02.03.074-1 | CMV Serology (IgG + IgM) | 9.80 | SIGTAP |
| 02.02.03.076-8; 02.02.03.087-3 | Toxoplasmosis Serology (IgG + IgM) | 15.40 | SIGTAP |
| 02.02.03.109-8 | Treponemal Test for Syphilis Detection | 1.78 | SIGTAP |
| 02.02.03.111-0 | Non-Treponemal Test for  Syphilis Detection (VDRL) | 1.23 | SIGTAP |
| 02.02.03.112-8 | FTA-ABS IgG Test for Syphilis | 4.33 | SIGTAP |
| 02.02.03.113-6 | FTA-ABS IgM Test for Syphilis | 4.33 | SIGTAP |
| 02.02.03.077-6; 02.02.03.088-1 | Chagas Serology (IgG + IgM antibody) | 8.04 | SIGTAP |
| 02.02.03.005-9 | Hepatitis C Virus RNA detection | 41.61 | SIGTAP |
| 03.06.02.001-7 | Therapeutic apheresis | 85.41 | SIGTAP |
| 03.06.02.006-8 | Red blood cell concentrate transfusion (outpatient) | 3.51 | SIGTAP |
| 03.06.02.006-8 | Red blood cell concentrate transfusion (inpatient) | 3.64 | SIGTAP |
| 03.06.02.007-6 | Platelet  Concentrate Transfusion (outpatient) | 3.51 | SIGTAP |
| 03.06.02.007-6 | Platelet  Concentrate Transfusion (inpatient) | 3.64 | SIGTAP |
| 03.04.10.001-3 | Treatment of clinical complications in cancer patients | 19.91 | SIGTAP |
| 03.04.10.001-3 | Day Clinic: Treatment of clinical complications in cancer patients (Day hospital or hospitalization): doctor/hospital | 19.91 | SIGTAP |
| 05.01.03.009-3 | Cryopreservation processing of bone marrow or peripheral blood HSC in Brazil for autogenic transplantation | 866.93 | SIGTAP |
| 05.01.03.007-7 | Mobilization, collection,  and preservation of Peripheral  Blood Stem Cells (PBSC) in Brazil | 1,066.86 | SIGTAP |
| 03.01.01.004-8 | Specialized care professional consultation (excluding doctor) | 2.73 | SIGTAP |
| 03.01.01.007-2 | Medical consultation in specialized care | 4.33 | SIGTAP |
| 02.02.03.023-7 | Immunophenotyping of malignant hemopathies (per marker) | 34.68 | SIGTAP |
| 04.06.02.007-8 | Insertion of semi or fully implantable long-term catheter | 185.80 | SIGTAP |
| 04.18.01.006-4 | Double-lumen catheter implant  for dialysis (SHILLEY®) | 50.20 | SIGTAP |
| NA | G-CSF (Filgrastim) | 15.04 | BPS |
| NA | Plerixafor  1 vial with 1.2 mL (20 mg/mL) | 7,493.88 | CMED - PF 0% ICMS |

Legend: All values were collected in February 2024. CEAC-Leste: State Clinical Analysis Center – East Zone - São Paulo State Secretariat – SP; SIGTAP: Table of Procedures, Drugs, Orthoses, Prostheses, and Special Materials of the SUS (*Sistema de Gerenciamento da Tabela de Procedimentos. Medicamentos e OPM do SUS*); BPS: Health Price Database; CMED: Drug Market Regulation Chamber; PF: factory price; ICMS: Tax on Operations related to the Circulation of Goods and Services on Interstate and Intermunicipal and Communication; CTH: hematopoietic stem cells; PCR: polymerase chain reaction; RNA: ribonucleic acid; CMV: cytomegalovirus; NA: not applicable; G-CSF, granulocyte colony-stimulating factor. All costs were presented in US dollars (USD) for 2024, converted from Brazilian Reais (BRL) using a cost conversion calculator based on purchasing power parity, available online (<https://eppi.ioe.ac.uk/costconversion/>).

**Supplementary Table 2 –** Resource consumption for cost estimation

| **Resource (measurement)** | **Unit price** | **Preemptive Plerixafor**  **n=82** | | **Rescue Plerixafor**  **n=203** | |
| --- | --- | --- | --- | --- | --- |
|  |  | **Consumed resource** | **Cost** | **Consumed resource** | **Cost** |
| **G-CSF** |  | 82 (100%) |  | 203 (100%) |  |
| **Vials** | US$ 15.04 | 20.00 (18.25 – 25.00)  22.37 ± 5.8 | US$ 300.82 (274.50 − 376.03)  US$ 336.41 ± 87.40 | 21.00 (16,00 – 30.00)  23.91 ± 9.5 | US$ 315.43  (240.66− 451.24)  US$ 359.62 ± 143.44 |
| **Days** |  | 5.00 (5.00 – 5.00)  5.10 ± 0.3 |  | 5.00 (5.00 – 6.00)  5.76 ± 1.7 |  |
| 4 |  | 0 |  | 23 (11.33%) |  |
| 5 |  | 71 (86.59%) |  | 132 (65.02%) |  |
| 6 |  | 11 (13.41%) |  | 10 (4.93%) |  |
| 9-11 |  | 0 |  | 38 (18.72%) |  |
| **Plerixafor** |  | 26 (31.7%) |  | 32 (15.8%) |  |
| **Vials** | US$ 7,493.88 | 0.00 (0.00 – 1.00)  0.35 ± 0.6 | US$ 0.00 (0.00 − 7,493.88)  US$ 2,650.27 ± 4,141.89 | 0.00 (0.00 – 0.00)  0.17 ± 0.4 | US$ 0.00 (0.00 − 0.00)  US$ 1,273.59 ± 3,309.07 |
| 1 |  | 23 (28.04%) |  | 26 (12.80%) |  |
| 2 |  | 3 (3.66%) |  | 6 (2.95%) |  |
| **Days** |  | 0.00 (0.00 – 1.00)  0.38 ± 0.6 |  | 0.00 (0.00 – 0.00)  0.18 ± 0.5 |  |
| 0 |  | 56 (68.29%) |  | 171 (84.24%) |  |
| 1 |  | 22 (26.83%) |  | 26 (12.81%) |  |
| 2 |  | 4 (4.88%) |  | 6 (2.96%) |  |
| **Complete blood count (unit)** | US$ 1.78 | 3.50 (2.00 – 5.00)  3.61 ± 1.9 | US$ 6.24 (3.56 – 8.91)  US$ 6.43 ± 3.38 | 3.00 (2.00 – 4.00)  3.63 ± 2.3 | US$ 5.34 (3.56 – 7.13)  US$ 6.46 ± 4.12 |
| **Biochemistry* (unit)** | US$ 3.99 | 12.00 (8.00 – 16.00)  12.46 ± 6.30 | US$ 8.73 (5.82 – 11.70)  US$ 9.00 ± 4.51 | 12.00 (8.00 – 17.00)  13.44 ± 8.00 | US$ 11.99 (7.99 – 16.93)  US$ 13.55 ± 8.06 |
| **Serologies** (unit)** | US$ 125.22 | 9.00 (8.00 – 16.00)  12.18 ± 4.9 | US$ 121.95 (112.10 – 224.20)  US$ 170.13 ± 68.14 | 16.00 (8.00 – 16.00)  13.93 ± 7.00 | US$ 224.20 (112.10 – 224.20)  US$ 194.53 ± 97.23 |
| **Coagulation painel† (unit)** | US$ 3.68 | 2.00 (1.00 – 2.00)  1.80 ± 0.90 | US$ 7.37 (3.68 – 7.37)  US$ 6.65 ± 3.29 | 2.00 (1.00 – 3.00)  2.06 ± 1.10 | US$ 7.37 (3.68 – 11.49)  US$ 7.60 ± 4.12 |
| **Beta-HCG (unit)** | US$ 3.40 | 0.00 (0.00 – 0.00)  0.10 ± 0.40 | US$ 0,00 (0,00 – 0,00)  US$ 0.43 ± 1.26 | 0.00 (0.00 – 0.00)  0.19 ± 0.70 | US$ 0.00 (0.00 – 0.00)  US$ 0.64 ± 2.51 |
| **Hepatitis B PCR (unit)** | US$ 72.16 | 0.00 (0.00 – 0.00)  0.04 ± 0.20 | US$ 0,00 (0,00 – 0,00)  US$ 2.67 ± 13.78 | 0.00 (0.00 – 0.00)  0.08 ± 0.30 | US$ 0.00 (0.00 – 0.00)  R$ 6.12 ± 22.71 |
| **Blood culture (unit)** | US$ 4.98 | 0.00 (0.00 – 0.00)  0.05 ± 0.30 | US$ 0,00 (0,00 – 0,00)  US$ 0.43 ± 1.73 | 0.00 (0.00 – 0.00)  0.12 ± 0.60 | US$ 0.00 (0.00 – 0.00)  US$ 0.59 ± 3.08 |
| **Medical consultation (unit)** | US$ 4.33 | 1.00 (1.00 – 1.00)  1.00 ± 0.00 | US$ 4.33 (4.33 – 4.33)  US$ 4.33 ± 0,00 | 1.00 (1.00 – 1.00)  1.19 ± 0.40 | US$ 4.33 (4.33– 4.33)  US$ 5.15 ± 1.69 |
| **Nursing consultation (unit)** | US$ 2.73 | 1.00 (1.00 – 1.00)  1.00 ± 0.00 | US$ 2.73 (2.73 – 2.73)  US$ 2.73 ± 0,00 | 1.00 (1.00 – 1.00)  1.19 ± 0.40 | US$ 2.73 (2.73 – 2.73)  US$ 3.37 ± 1.52 |
| **Hospital day care (days)** | US$ 19.91 | 3.00 (3.00 – 4.00)  3.28 ± 0.90 | US$ 59.73 (59.73 – 79.64)  US$ 65.31 ± 18.86 | 3.00 (3.00 – 4.00)  3.66 ± 1.40 | US$ 59.73(59.73 – 79.64)  US$ 72.87 ± 27.70 |
| **Inpatient care (days)** | US$ 19.91 | 0.00 (0.00 – 2.00)  0.91 ± 1.40 | US$ 0.00 (0.00 – 34.68)  US$ 15.86 ± 24.23 | 0.00 (0.00 – 0.00)  0.64 ± 1.40 | US$ 0.00 (0.00 – 0.00)  US$ 14.20 ± 33.77 |
| **HSC cryopreservation (days)** | US$ 866.93 | 1.00 (1.00 – 1.00)  1.16 ± 0.40 | US$ 866.93 (866.93 – 866.93)  US$ 1,004.37 ± 318.60 | 1.00 (1.00 – 1.00)  1.06 ± 0.60 | US$ 866.93 (866.93 – 866.93)  US$ 918.18 ± 477.55 |
| **PB CD34+ count - flow cytometry (unit)** | US$ 34.68 | 1.00 (1.00 – 1.00)  1.04 ± 0.20 | US$ 34.68 (34.68 – 34.68)  US$ 35.95 ± 6.55 | 1.00 (1.00 – 1.00)  1.28 ± 0.60 | US$ 34.68 (34.68 – 34.68)  US$ 44.24 ± 19.29 |
| **HSC collections and storage (days)** | US$ 1,066.86 | 1.00 (1.00 – 1.00)  1.17 ± 0.40 | US$ 1,066.86 (1,066.86 – 1,066.86)  US$ 1,249.00 ± 403.90 | 1.00 (1.00 – 1.00)  1.04 ± 0.50 | US$ 1,066.86 (1,066.86 – 1,066.86)  US$ 1,111.53 ± 558.69 |
| **Red blood cells and platelets transfusion** **(unit)** | US$ 3.64 | 0.00 (0.00 – 0.00)  0.06 ± 0.20 | US$ 0,00 (0,00 – 0,00)  US$ 0.43 ± 0.82 | 0.00 (0.00 – 0.00)  0.08 ± 0.30 | US$ 0.00 (0.00 – 0.00)  US$ 0.43 ± 1.17 |
| **CVC (unit)** | **Double-lumen dialysis catheter:** US$ 50.20  **Semi-permanent long-term implantable:** US$185.80 | 1.00 (1.00 – 1.00)  0.91 ± 0.30 | US$ 50.20 (50.20 – 50.20)  US$ 78.99 ± 62.59 | 1.00 (0.50 – 1.00)  0.76 ± 0.50 | US$ 50.20 (0.43 – 115.81)  US$ 31.95 ± 25.14 |

Legend: Data presented as n (%), median (interquartile range), or mean ± standard deviation; unit prices are for the year 2024; *Biochemistry - a combination of tests: potassium, ionized calcium, magnesium, creatinine;**Serologies - a combination of tests: hepatitis B, hepatitis C, HIV 1 and 2, HTLV1 and 2, syphilis, Chagas disease, toxoplasmosis, cytomegalovirus; † Coagulation panel - a combination of tests: prothrombin time and activated partial thromboplastin time; G-CSF, granulocyte colony-stimulating factor; Beta-HCG, Beta-human chorionic gonadotropin; PCR, polymerase chain reaction; CD, the cluster of differentiation; PB, peripheral blood; CVC, central venous catheter; HSC, Hematopoietic Stem Cell. Values in Brazilian Reais from 2024 converted to US Dollars in the same year using the CCEMG - EPPI-Centre Cost Converter (v.1.6) - <https://eppi.ioe.ac.uk/costconversion/>.

**Supplementary Table 3 –** Sensitivity Analysis by Varying the Average Cost per Patient in Each Strategy

| **Clinical outcomes** | **Base case** | **Lower bound** | **Upper bound** |
| --- | --- | --- | --- |
| **Average cost per patient*** |  |  |  |
| Preemptive plerixafor | US$ 5,642.54 | US$ 4,650.18 | US$ 6,634.91 |
| Rescue plerixafor | US$ 4,110.11 | US$ 3,566.87 | US$ 4,653.35 |
| **ICER: % collections ≥2 × 10^6^/kg CD34+ cells** |  |  |  |
| Preemptive plerixafor | US$ 151.28 | US$ 53.32 | US$ 294.24 |
| Rescue plerixafor | US$ 151.28 | US$ 204.90 | US$ 97.65 |
| **ICER: % Progression to ASCT** |  |  |  |
| Preemptive plerixafor | US$ 116.18 | US$ 40.94 | US$191.42 |
| Rescue plerixafor | US$ 116.18 | US$ 157.37 | US$ 75.00 |
| **ICER: % collections ≥4 × 10^6^/kg CD34+ cells** |  |  |  |
| Preemptive plerixafor | US$ 326.05 | US$ 114.91 | R$ 1,239.30 |
| Rescue plerixafor | US$ 326.05 | US$ 441.63 | US$ 210.47 |
| **ICER: Nº leukapheresis sessions** |  |  |  |
| Preemptive plerixafor | -US$ 15,324.36 (dominated) | -US$ 5,400.74 (dominated) | - US$ 25,247.99  (dominated) |
| Rescue plerixafor | -US$ 15,324.36 (dominated) | -US$ 20,756.74 (dominated) | -US$ 9,891.98 (dominated) |

Legend: *The average cost per patient in each strategy with the respective 95% confidence interval varied in the estimates of ICER while keeping each clinical outcome at the value of the baseline analysis. ICER, incremental cost-effectiveness ratio; ASCT Autologous Stem Cell Transplantation; N= number. Values in Brazilian Reais from 2024 converted to US Dollars in the same year using the CCEMG - EPPI-Centre Cost Converter (v.1.6) - <https://eppi.ioe.ac.uk/costconversion/>.

**Supplementary Table 4** – Sensitivity analysis by varying the price of filgrastim

| **Clinical Outcomes** | **Base** | **Lower bound** | **Upper bound** |
| --- | --- | --- | --- |
| **Cost per patient (mean)*** |  |  |  |
| Preemptive plerixafor | US$ 5,642.54 | US$ 5,596.01 | US$ 6,451.09 |
| Rescue plerixafor | US$ 4,110.24 | US$ 4,060.36 | US$ 4,974.43 |
| **ICER – % collections ≥2 × 10^6^/kg CD34+ cells** |  |  |  |
| Preemptive plerixafor | US$ 151.27 | US$ 146.67 | US$ 231.08 |
| Rescue plerixafor | US$ 151.27 | US$ 156.19 | US$ 65.95 |
| **ICER – % Progression to ASCT** |  |  |  |
| Preemptive plerixafor | US$ 116.17 | US$ 112.64 | US$ 177.47 |
| Rescue plerixafor | US$ 116.17 | US$ 119.95 | US$ 50.65 |
| **ICER – % collections ≥ 4 × 10^6^/kg CD34+ cells** |  |  |  |
| Preemptive plerixafor | US$ 326.02 | US$ 316.12 | US$ 498.05 |
| Rescue plerixafor | US$ 326.02 | US$ 332.30 | US$ 142.15 |
| **ICER –Leukapheresis sessions numbers** |  |  |  |
| Preemptive plerixafor | -US$ 15,323.02 (dominated) | -US$ 14,857.69 (dominated) | -US$ 23,408.45 (dominated) |
| Rescue plerixafor | -US$ 15,323.02  (dominated) | -US$ 15,821.80 (dominated) | -US$ 6,681.10 (dominated) |

Legend: *The average cost per patient in each strategy varied according to the minimum and maximum price of filgrastim (US$ 12.96 - US$ 51.19) in the estimates of ICER while keeping each clinical outcome at the value of the baseline analysis; ICER, incremental cost-effectiveness ratio; ASCT, Autologous Stem Cell Transplantation; CD, cluster of differentiation. Values in Brazilian Reais from 2024 converted to US Dollars in the same year using the CCEMG - EPPI-Centre Cost Converter (v.1.6) - <https://eppi.ioe.ac.uk/costconversion/>.

**Supplementary Table 5 –** Sensitivity Analysis by Variation in Clinical Outcomes

| **Clinical Outcomes** | **Parameter** | | |  | **ICER** | | |
| --- | --- | --- | --- | --- | --- | --- | --- |
|  | **Base case** | **95% lower confidence bound** | **95% upper confidence bound** |  | **Base case** | **Lower bound** | **Upper bound** |
| **% collections ≥2 × 10^6^/kg CD34+ cells** |  |  |  |  |  |  |  |
| Preemptive  plerixafor | 96.34 | 88.94 | 99.05 |  | US$ 151.28 | US$ 561.33 | US$ 119.35 |
| Rescue plerixafor | 86.21 | 80.51 | 90.49 |  | US$ 151.28 | US$ 96.81 | US$ 261.95 |
| **% Progression to ASCT** |  |  |  |  |  |  |  |
| Preemptive  plerixafor | 86.59 | 76.85 | 92.79 |  | US$ 116.18 | US$ 448.18 | US$ 79,03 |
| Rescue plerixafor | 73.4 | 66,67 | 79.23 |  | US$ 116.18 | US$ 76.93 | US$ 208.21 |
| **% collections ≥4 × 10^6^/kg CD34+ cells** |  |  |  |  |  |  |  |
| Preemptive  plerixafor | 47.6 | 36.53 | 58.82 |  | US$ 326.05 | -US$ 240.57  (dominated) | US$ 96.26 |
| Rescue plerixafor | 42.9 | 36.01 | 49.98 |  | US$ 326.05 | US$ 132.22 | - US$ 643.88  (dominated) |
| **Nº leukapheresis sessions** |  |  |  |  |  |  |  |
| Preemptive  plerixafor | 1.17 | 1.09 | 1.25 |  | -US$ 15,324.36  (dominated) | -US$ 76,621.81 (dominated) | -US$ 8,513.53  (dominated) |

Legend: The 95% confidence interval (95% CI) was used for clinical variables. In all analyses, the average cost per patient from the base case analysis was maintained (preemptive plerixafor US$ 5,642.54 and rescue plerixafor US$ 4,110.11), ICER: incremental cost-effectiveness ratio; NA: not applicable; ASCT: autologous stem cell transplant; CD, a cluster of differentiation. Values in Brazilian Reais from 2024 converted to US Dollars in the same year using the CCEMG - EPPI-Centre Cost Converter (v.1.6) - <https://eppi.ioe.ac.uk/costconversion/>.

**Supplementary Table 6** – Estimation of the Mean Incremental Cost-Effectiveness Ratio and Confidence Interval by Bootstrapping

| **Clinical outcomes** | **Mean** | **95% CI*** |
| --- | --- | --- |
| **% collections ≥2 ×10^6^/kg CD34+ (minimum collection)** | US$ 193.41 | US$ 35.93 to US$ 549.61 |
| **% Progression to ASCT** | US$ 155.81 | US$ 26.37 to US$ 473.68 |
| **% collections ≥4 ×10^6^/kg CD34+ (optimal collection)** | US$ 196.57 | -US$ 2,633.55 to US$ 3,040.85 |
| **N° leukapheresis sessions (mean)** | -US$ 16,991.61 | -US$ 67,397.44 to US$ 10,415.28 |

Legend: Estimates calculated from 10,000 bootstraps resamples, * 95% CI estimate based on the 2.5th and 97.5th percentiles of the simulations; 95% CI, 95% confidence interval; ASCT, autologous stem cell transplant; CD, cluster of differentiation. Values in Brazilian Reais from 2024 converted to US Dollars in the same year using the CCEMG - EPPI-Centre Cost Converter (v.1.6) - <https://eppi.ioe.ac.uk/costconversion/>.

**Supplementary Table 7** – Demographic, clinical characteristics, clinical outcomes and cost estimates per disease

| **Variable** | **MM** | | |  | **Lymphomas** | | |
| --- | --- | --- | --- | --- | --- | --- | --- |
|  | **Preemptive plerixafor**  **n = 56** | **Rescue plerixafor**  **n = 140** | ***p-value** |  | **Preemptive plerixafor**  **n = 26** | **Rescue plerixafor**  **n = 63** | ***p-value** |
| **Age, years** | 58.5 (54.75 ­ 63.00)  57.66 ± 8.06 | 60.00 (54.00 – 65.25)  58.66 ± 8.96 | 0.311 |  | 47.00 (28.00 – 56.00)  42.88 ± 15.99 | 42.00 (30.00 – 56.50)  42,84 ± 14.21 | 0.989 |
| **Male** | 26 (46.4%) | (46.4%) | 0.9 |  | 12 (46.2%) | 33 (52.4%) | 0.593 |
| **Race** |  |  | 0.862 |  |  |  | 0.699 |
| White | 31 (55.4%) | 79 (56.4%) |  |  | 17 (65.4%) | 35 (55.6%) |  |
| Mixed | 21 (37.5%) | 48 (34.3%) |  |  | 8 (30.7%) | 223 (35.5%) |  |
| Black | 4 (7.2%) | 13 (9.3%) |  |  | 1 (3.8%) | 5 (7.9%) |  |
| **Staging** |  |  | 0.644 |  |  |  | 0.441 |
| I | 5 (10%) | 8 (6.4%) |  |  | 1 (4%) | 2 (3.3%) |  |
| II | 18 (36%) | 39 (31%) |  |  | 8 (32%) | 12 (19.7%) |  |
| III | 27 (54%) | 77 (61.6%) |  |  | 15 (20%) | 10 (16.4%) |  |
| IV | 0 | 1 (0.8%) |  |  | 11 (44%) | 37 (60.7%) |  |
| Missing | 6 | 15 |  |  | 1 | 2 |  |
| **PB CD34+ D4** | 21.08 (11.60 -34.25)  26 ± 21.61 | 14.85 (5.90 - 27.62)  27.17 ± 40.51 | 0.085 |  | 13.50 (4.78 – 24.70)  23.1 ± 28.2 | 10.00 (3.66 – 24.35)  19.7 ± 24.1 | 0.499 |
| **collection yield**  **(x10^6^ CD34+/kg)** | 4.24 (2.79 – 6.3)  5.08 ± 2.96 | 3.81 (2.53 – 6.17)  4.61 ± 3.22 | 0.302 |  | 3.17 (2.35 – 5.10)  4.1 ± 2.3 | 3.00 (2.05 – 5.03)  3.8 ± 3.0 | 0.430 |
| **Minimum collection** | 54 (96.4%) | 125 (89.3%) | 0.160 |  | 25 (96.2%) | 50 (79.4%) | 0.058 |
| **Optimal collection** | 29 (51.8%) | 67 (47.9%) | 0.619 |  | 9 (34.6%) | 20 (31.8%) | 0.793 |
| **ASCT progression** | 47 (83.9%) | 112 (80%) | 0.525 |  | 21 (80.8%) | 37 (58.7%) | 0.047 |
| **Leukapheresis sessions** | 1.00 (1.00 -1.00)  2.18 ± 0.39 | 1.00 (1.00 – 1.00)  2.06 ± 0.5 | 0.110 |  | 1.00 (1.00 -1.00)  2.2 ± 0.4 | 1.00 (1.00 -1.00)  2.1 ± 0.7 | 0.486 |
| **Days between first mobilization and ASCT** | 28.00 (7.50 – 102.00)  67.5 ± 73.1 | 90.00 (36.25 – 150.00)  112.4 ± 126.7 | 0.003 |  | 57.00 (15.00 – 120.00)  76.6 ± 78.8 | 103.00 (49.00 – 187.50)  149.9 ± 149.5 | 0.018 |
| **Remobilization** | 0 | 22 (15.7%) | 0.002 |  | 0 | 16 (25.4%) | 0.004 |
| **Days between mobilization and remobilization** | 0 .00 (0.00-0.00)  0 | 0 .00 (0.00-0.00)  21.4 ± 66.2 | 0.002 |  | (0.00 -0.00)   1. 0 | (0.00 -0.00)  32.3 ± 76.7 | 0.007 |
| **Total cost/patient,**  **US$** | 2,729.40 (2,554.19 – 6,205.31)  5,008.53 ± 4,009.04 | 2,643.62 (2,499.97 – 4,434.94)  3,859.22 ± 3,327.87 | 0.043 |  | 3,799.29 (2,640.45 – 10,190.07)  7,008.12 ± 5,281.10 | 2,693.30 (2,504.70 – 4,651.27)  4,676.24 ± 4,983.94 | 0.052 |

Legend: Date presented as n (%), median (interquartile range), mean ± standard deviation. *Pearson’s Chi-squared test; Fisher’s exact test; Wilcoxon rank sum test. ASCT, autologous stem cell transplantation; CD, Cluster of differentiation; D4, day 4; MM, Multiple Myeloma; PB, peripheral blood; US$, US Dollars. Values in Brazilian Reais from 2024 converted to US Dollars in the same year using the CCEMG - EPPI-Centre Cost Converter (v.1.6) - <https://eppi.ioe.ac.uk/costconversion/>.

**Supplementary Table 8** – Incremental Cost-Effectiveness Ratios for Multiple Myeloma

| **Variable** | **Preemptive plerixafor**  **n = 56** | **Rescue Plerixafor**  **n = 140** | **Difference (∆)** | **ICER**  **(∆ cost/ ∆ outcome)** |
| --- | --- | --- | --- | --- |
| **Cost (mean), US$** | 5,008.53 | 3,859.22 | 1,149.31 |  |
| **% minimum collection** | 96.43 | 89.29 | 7,14 | US$ 160,97 |
| **% ASCT** | 83.93 | 80.00 | 3.93 | US$ 292.45 |
| **% optimal collection** | 51.79 | 47.86 | 3.93 | US$ 292.45 |
| **Leukapheresis sessions (mean)** | 2.18 | 2.06 | 0.12 | -US$ 9,577.58  Dominated * |

Legend: *Incremental cost-effectiveness ratio (ICER) per avoided leukapheresis session; ICER, incremental cost-effectiveness ratio; ASCT, autologous stem cell transplantation; ∆, the difference between preemptive and rescue group; CD, Cluster of differentiation; US$, US Dollars. Values in Brazilian Reais from 2024 converted to US Dollars in the same year using the CCEMG - EPPI-Centre Cost Converter (v.1.6) - <https://eppi.ioe.ac.uk/costconversion/>

**Supplementary Table 9** – Incremental Cost-Effectiveness Ratios for Lymphomas (Hodgkin and Non-Hodgkin)

| **Variable** | **Preemptive plerixafor**  **n = 26** | **Rescue Plerixafor**  **n = 63** | **Difference (∆)** | **ICER**  **(∆ cost/ ∆ outcome)** |
| --- | --- | --- | --- | --- |
| **Cost (mean), US$** | 7,008.12 | 4,676.24 | 2,331.88 |  |
| **% minimum collection** | 96.15 | 79.37 | 16.78 | US$ 138.97 |
| **% ASCT** | 80.77 | 58.73 | 22.04 | US$ 105.80 |
| **% optimal collection** | 34.62 | 31.75 | 2.87 | US$ 812.50 |
| **Leukapheresis sessions (mean)** | 2.15 | 2.08 | 0.07 | - US$ 33,312.57  Dominated * |

Legend: *Incremental cost-effectiveness ratio (ICER) per avoided leukapheresis session; ICER, incremental cost-effectiveness ratio; ASCT, autologous stem cell transplantation; ∆, the difference between preemptive and rescue group; CD, Cluster of differentiation; US$, US Dollars. Values in Brazilian Reais from 2024 converted to US Dollars in the same year using the CCEMG - EPPI-Centre Cost Converter (v.1.6) - <https://eppi.ioe.ac.uk/costconversion/>

**Supplementary Table 10** – Demographic, clinical characteristics, clinical outcomes, and cost estimates for poor mobilizers, overall and per disease

|  | **Overall** | | | **MM** | | | **Lymphomas** | | |
| --- | --- | --- | --- | --- | --- | --- | --- | --- | --- |
| **Variable** | **Preemptive plerixafor**  **n = 23** | **Rescue plerixafor**  **n = 74** | ***p-value** | **Preemptive plerixafor**  **n = 13** | **Rescue plerixafor**  **n = 43** | ***p-value** | **Preemptive plerixafor**  **n = 10** | **Rescue plerixafor**  **n = 31** | ***p-value** |
| **Age, years** | 59.00 (40.00 – 63.00)  51.3 ± 15.96 | 59.50 (50.00 – 63.00)  53.91 ± 13.44 | 0.440 | 63.00 (58.00 – 65.00)  60.54 ± 5.65 | 61.00 (57.00 – 66.50)  59.86 ± 9.34 | 0.805 | 33.00 (25.75 – 56.75)  39.3 ± 17.22 | 50.00 (32.00 – 60.00)  45.65 ± 14.01 | 0.246 |
| **Male** | 8 (34.8%) | 39 (52.7%) | 0.157 | 5 (38.5%) | 24 (55.8%) | 0.349 | 3 (30%) | 15 (48.4%) | 0.467 |
| **Race** |  |  | 0.644 |  |  | 0.851 |  |  | 1.000 |
| White | 13 (56.5%) | 43 (58.1%) |  | 7 (53.8%) | 26 (60.5%) |  | 6 (60%) | 17 (54.8%) |  |
| Mixed | 10 (43.5%) | 27 (36.5%) |  | 6 (46.2%) | 15 (34.9%) |  | 4 (40%) | 12 (38.7%) |  |
| Black | 0 | 4 (5.4%) |  | 0 | 2 (4.7%) |  | 0 | 2 (6.5%) |  |
| **Staging** |  |  | 0.809 |  |  | 0.745 |  |  | 0.557 |
| I | 0 | 3 (4.1%) |  | 0 | 2 (4.7%) |  | 0 | 1 (3.2%) |  |
| II | 6 (26.1%) | 18 (24.3%) |  | 2 (15.4%) | 12 (27.9%) |  | 4 (40%) | 6 (19.4%) |  |
| III | 12 (52.2%) | 29 (39.2%) |  | 10 (76.9%) | 24 (55.8%) |  | 2 (20%) | 5 (16.1%) |  |
| IV | 4 (17.4%) | 19 (25.7%) |  | 0 | 0 |  | 4 (40%) | 19 (61.3%) |  |
| Missing | 1 (4.3%) | 5 (6.8%) |  | 1 (7.7%) | 5 (11.6%) |  | 0 | 0 |  |
| **PB CD34+ D4** | 4.30 (2.05 – 6.55)  4.44 ± 2.84 | 3.62 (2.02 – 6.00)  3.95 ± 2.56 | 0.431 | 4.30 (2.00 – 7.10)  4.36 ± 2.96 | 3.90 (2.20 – 5.34)  3.87 ± 2.48 | 0.556 | 4.25 (2.92 – 5.90)  4.56 ± 2.83 | 3.33 (2.00 – 6.00)  4.06 ± 2.7 | 0.616 |
| **Collection yield**  **(x10^6^ CD34+/kg)** | 3.00 (2.28 – 3.93)  3.29 ± 1.47 | 2.46 (0.00 – 3.37)  2.6 ± 2.67 | 0.236 | 3.20 (2.10 – 3.69)  3.26 ± 1.5 | 2.67 (0.00 – 4.25)  3.16 ± 3.16 | 0.908 | 2.52 (2,30 – 4.04)  3.33 ± 1.51 | 2.18 (0.00 – 2.89)  1.82 ± 1.50 | 0.009 |
| **Minimum collection** | 20 (87%) | 48 (64.9%) | 0.066 | 11 (84.6%) | 29 (67.4%) | 0.308 | 9 (90%) | 19 (61.3%) | 0.129 |
| **Optimal collection** | 7 (30.4%) | 14 (18.9%) | 0.257 | 4 (30.8%) | 12 (27.9%) | 1 | 3 (30%) | 2 (6.5%) | 0.083 |
| **ASCT progression** | 17 (73.9%) | 41 (55.4%) | 0.351 | 8 (61.5%) | 25 (58.1) | 1.000 | 9 (90%) | 16 (51.6%) | 0.074 |
| **Leukapheresis sessions** | 1.00 (1.00 -1.50)  1.26 ± 0.45 | 1.00 (0.00 -1.00)  0.92 ± 0.74 | 0.038 | 1.00 (1.00 – 2.00)  1.31 ± 0.48 | 1.00 (0.00 – 1.00)  0.84 ± 0.61 | 0.014 | 1.00 (1.00 – 1.00)  1.20 ± 0.42 | 1.00 (0.00 – 2.00)  1.03 ± 0.87 | 0.564 |
| **Days between first mobilization and ASCT** | 15.00 (4.00 – 50.00)  38.53 ± 55.96 | 124.50 (48.00 – 213.75)  137.45 ± 108.84 | 0.001 | 14.50 (4.00 – 57.50)  36.12 ± 43.8 | 97.00 (30.00 – 170.00)  120.60 ± 99.03 | 0.027 | 15.00 (5.00 – 25.00)  40.67 ± 67.63 | 150.00 (60.00 – 245.00)  165.53 ± 121.80 | 0.01 |
| **Remobilization** | 0 | 36 (48.6%) | <0.001 | 0 | 21 (48.8%) | 0.001 | 0 | 15 (48.4%) | 0.007 |
| **Days between mobilization and remobilization** | 0,00 (0.00 -0.00)  0 | 0.00 (0.00 – 99.00)  67.43 ± 102.45 | 0.002 | 0.00 (0.00 -0.00)  0 | 0.00 (0.00 – 105.00)  69.37 ± 105.33 | 0.022 | 0.00 (0.00 – 0.00)  0 ± 0 | 0.00 (0.00 – 95.50)  64.74 ± 99.97 | 0.049 |
| **Total cost/patient,**  **US$** | 10,236.96 (10,168.50 - 11,198.96)  11,692.20 ± 3,274.93 | 3,829.85 (605.17 - 10,557.74)  6,146.40 ± 5,881.01 | <0.001 | 10,268.81 (10,188.22 – 12,062.62)  11,396.10 ± 2,724.53 | 3,191.34 (604.06 – 10,556.94)  5,903.06 ± 5,357.00 | 0.001 | 10,199.12 (10,130.64 – 10,306.42)  12,077.14 ± 4,003.55 | 4,456.23 (625.30 – 10,557.11)  6,483.94 ± 6,616.71 | 0.016 |

Legend: Date presented as n (%), median (interquartile range), mean ± standard deviation. *Pearson’s Chi-squared test; Fisher’s exact test; Wilcoxon rank sum test. ASCT, autologous stem cell transplantation; CD, Cluster of differentiation; D4, day 4; MM, Multiple Myeloma; PB, peripheral blood; US$, US Dollars. Values in Brazilian Reais from 2024 converted to US Dollars in the same year using the CCEMG - EPPI-Centre Cost Converter (v.1.6) - <https://eppi.ioe.ac.uk/costconversion/>.

**Supplementary Table 11** – Incremental Cost-Effectiveness Ratios for Poor Mobilizers – Overall

| **Variable** | **Preemptive plerixafor**  **n = 24** | **Rescue Plerixafor**  **n = 74** | **Difference (∆)** | **ICER**  **(∆ cost/ ∆ outcome)** |
| --- | --- | --- | --- | --- |
| **Cost (mean), US$** | 11,692.20 | 6,146.40 | 5,545.80 |  |
| **% minimum collection** | 87 | 64.9 | 22.1 | US$ 250.94 |
| **% ASCT** | 73.9 | 55.4 | 18.5 | US$ 299.77 |
| **% optimal collection** | 30.4 | 18.9 | 11.5 | US$ 482.24 |
| **Leukapheresis sessions (mean)** | 1.26 | 0.92 | 0.34 | - US$ 16,311.18  Dominated* |

Legend: *Incremental cost-effectiveness ratio (ICER) per avoided leukapheresis session; ICER, incremental cost-effectiveness ratio; ASCT, autologous stem cell transplantation; ∆, the difference between preemptive and rescue group; CD, Cluster of differentiation; US$, US Dollars. Values in Brazilian Reais from 2024 converted to US Dollars in the same year using the CCEMG - EPPI-Centre Cost Converter (v.1.6) - <https://eppi.ioe.ac.uk/costconversion/>

**Supplementary Table 12** – Incremental Cost-Effectiveness Ratios for Poor Mobilizers – Multiple Myeloma

| **Variable** | **Preemptive plerixafor**  **n =13** | **Rescue Plerixafor**  **n = 43** | **Difference (∆)** | **ICER**  **(∆ cost/ ∆ outcome)** |
| --- | --- | --- | --- | --- |
| **Cost (mean), US$** | 11,396.10 | 5,903.06 | 5,493.04 |  |
| **% minimum collection** | 84.6 | 67.4 | 17.2 | US$ 319.36 |
| **% ASCT** | 61.5 | 58.1 | 3.4 | US$ 1,615.6 |
| **% optimal collection** | 30.8 | 27.9 | 2.9 | US$ 1,894.15 |
| **Leukapheresis sessions (mean)** | 1.31 | 0.84 | 0.47 | - US$ 11,687.32  Dominated* |

Legend: *Incremental cost-effectiveness ratio (ICER) per avoided leukapheresis session; ICER, incremental cost-effectiveness ratio; ASCT, autologous stem cell transplantation; ∆, the difference between preemptive and rescue group; CD, Cluster of differentiation; US$, US Dollars. Values in Brazilian Reais from 2024 converted to US Dollars in the same year using the CCEMG - EPPI-Centre Cost Converter (v.1.6) - <https://eppi.ioe.ac.uk/costconversion/>

**Supplementary Table 13** – Incremental Cost-Effectiveness Ratios for Poor Mobilizers – Lymphomas (Hodgkin and Non-Hodgkin)

| **Variable** | **Preemptive plerixafor**  **n = 11** | **Rescue Plerixafor**  **n = 31** | **Difference (∆)** | **ICER**  **(∆ cost/ ∆ outcome)** |
| --- | --- | --- | --- | --- |
| **Cost (mean), US$** | 12,077.14 | 6,483.94 | 5,593.20 |  |
| **% minimum collection** | 90 | 61.3 | 28.7 | US$ 194.89 |
| **% ASCT** | 90 | 51.6 | 38.4 | US$ 145.66 |
| **% optimal collection** | 30 | 6.5 | 23.5 | US$ 238.01 |
| **Leukapheresis sessions (mean)** | 1.20 | 1.03 | 0.17 | - US$ 32,901.18  Dominated* |

Legend: *Incremental cost-effectiveness ratio (ICER) per avoided leukapheresis session; ICER, incremental cost-effectiveness ratio; ASCT, autologous stem cell transplantation; ∆, the difference between preemptive and rescue group; CD, Cluster of differentiation; US$, US Dollars. Values in Brazilian Reais from 2024 converted to US Dollars in the same year using the CCEMG - EPPI-Centre Cost Converter (v.1.6) - <https://eppi.ioe.ac.uk/costconversion/>
